# Updated cranial and mandibular description of the Late Cretaceous (Maastrichtian) baenid turtle *Saxochelys gilberti* based on micro-computed tomography scans and new information on the holotype-shell association

**DOI:** 10.1186/s13358-023-00301-6

**Published:** 2024-01-23

**Authors:** Gaël E. Spicher, Tyler R. Lyson, Serjoscha W. Evers

**Affiliations:** 1 Departement of Geosciences, University of Fribourg, 1700 Fribourg, Switzerland; 2 Institute of Geosciences, Section Paleontology, Rheinische Friedrich-Wilhelms-Universität Bonn, Nussallee 8, 53115 Bonn, Germany; 3 Department of Earth Sciences, Denver Museum of Nature & Science, Denver, CO USA

**Keywords:** Late Cretaceous, Hell Creek Formation, Testudinata, Paracryptodira, Baenidae, Digital anatomy

## Abstract

*Saxochelys gilberti* is a baenid turtle from the Late Cretaceous Hell Creek Formation of the United States of America known from cranial, shell, and other postcranial material. Baenid turtles are taxonomically diverse and common fossil elements within Late Cretaceous through Eocene faunas. Detailed anatomical knowledge is critical to understanding the systematics and morphological evolution of the group. This is particularly important as baenids represent an important group of continental vertebrates that survived the mass extinction event associated with the Cretaceous/Paleogene boundary. High-resolution micro-computed tomography scanning of the holotype skull reveals additional anatomical details for the already well-known *Saxochelys gilberti*. This includes the revision of some anatomical statements from the original description, but also detailed knowledge on internal anatomical features of the braincase and the description of a well-preserved axis (cervical vertebra 2). Our new detailed description and previous work on the shell and postcrania make *Saxochelys* one of the best-described, nearly complete baenid turtles, which are often only known from either isolated shell or cranial material. A revised phylogenetic analysis confirms the position of *Saxochelys gilberti* as a derived baenid (Eubaeninae) more closely related to *Baena arenosa* than to *Eubaena cephalica*.

**Supplementary Information:**

The online version contains supplementary material available at 10.1186/s13358-023-00301-6.

## Introduction

Baenidae is a clade of paracryptodiran turtles that lived in freshwater environments on the North American continent from the Early Cretaceous to the Eocene (Hay, 1908; Gaffney, 1972a; Hutchison 1984; Sullivan et al., 1988; Joyce & Lyson, 2015). The Hell Creek Formation is particularly well-known for its extraordinary fossil record (e.g., Hartman et al., 2002; Wilson et al., 2014), especially for vertebrates. The turtle fauna of the Hell Creek Formation is singularly rich and taxonomically diverse (e.g., Hay, 1902; Whetstone, 1978; Meylan & Gaffney, 1989; Holroyd & Hutchison, 2002; Knauss et al., 2011; Joyce & Lyson, 2011; Lyson & Joyce, 2011; Vitek, 2012; Holroyd et al., 2014; Jasinski et al., 2022), and baenid turtles compose the major part of it with the presence of at least 12 taxa (Holroyd et al., 2014; Lyson et al., 2019). According to Joyce & Lyson (2015), the diversity of Baenidae was highest during the Maastrichtian (Late Cretaceous) and the Danian (Early Paleogene). A common topic of interest is the survival of baenid turtles across the Cretaceous/Paleogene (K/Pg) boundary (Hutchison & Archibald, 1986; Lyson & Joyce, 2009a, 2015; Lyson et al., 2011; Holroyd et al., 2014; Jasinski et al., 2018; Adrain, 2022), which is associated with an asteroid-impact driven mass extinction event (e.g., Chiarenza et al., 2020; Fastovsky & Sheehan, 2005; Hull et al., 2020; Raup & Sepkoski, 1982; Schulte et al., 2010). To understand survivorship, including the potential selective survivorship of ecological guilds (e.g., Evers & Joyce, 2020; Jasinski et al., 2018; Lyson & Joyce, 2009a), the communities on either side of the extinction event should be known well. *Saxochelys gilberti* from the Maastrichtian of the Hell Creek Formation described by Lyson et al. (2019) is one taxon participating in the high diversity of baenid turtles of this formation. We herein describe the holotype cranium, mandibular, hyoid, and anterior cervical vertebral material of *Saxochelys gilberti* (DMNH EPV. 96000; Denver Museum of Nature & Science, Denver, Colorado, U.S.A.), using a micro-computed tomography (µCT) scan, correcting some previous anatomical statements that were in error and providing otherwise more detail on the previously known anatomy.

## Materials and methods

### New information on the association of a shell with the holotype skull

**Fig. 1:**
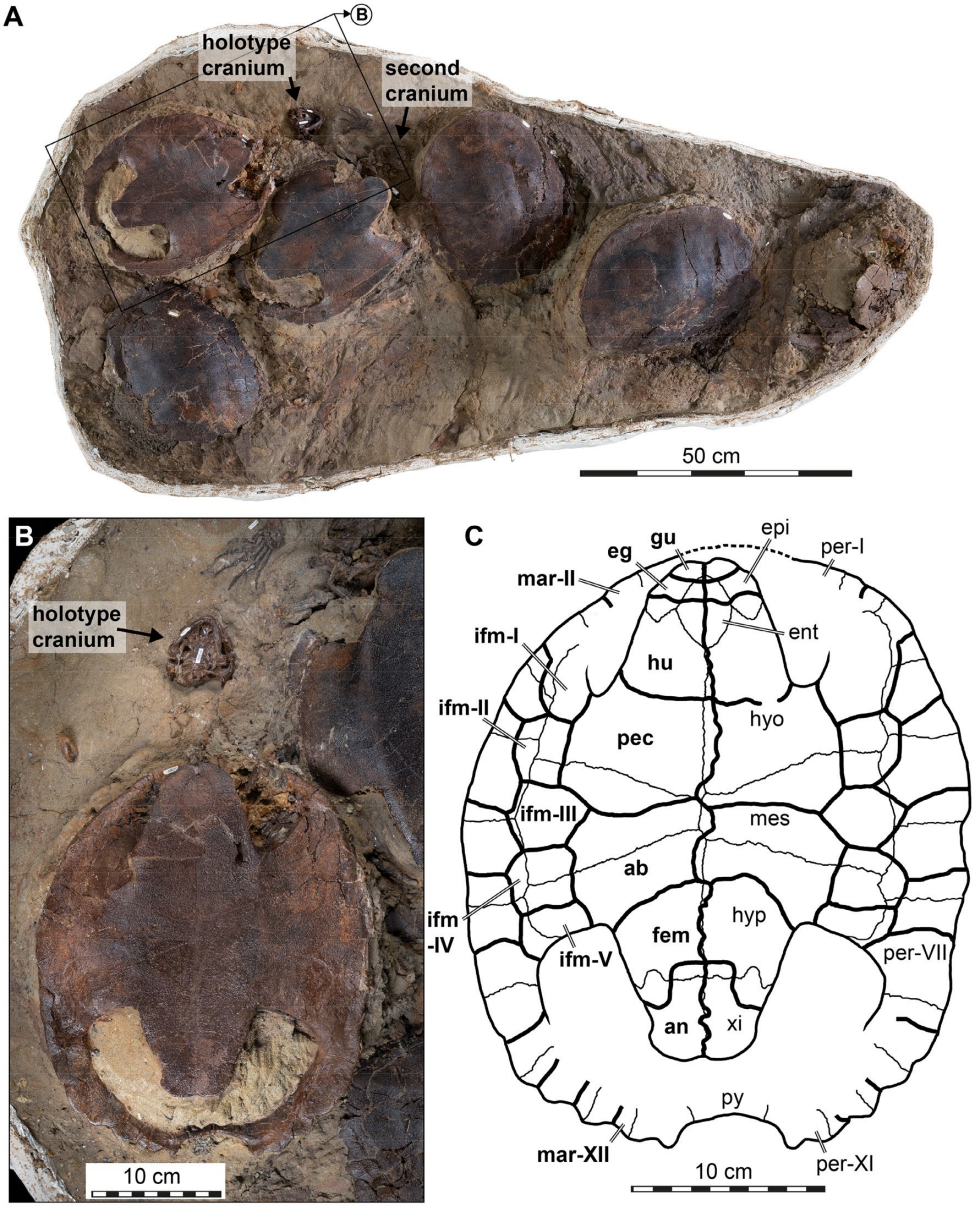
Photographs of the associated fossils of the holotype skull of *Saxochelys gilberti* (DMNH EPV.96000), showing its associated shell and an interpretative line drawing. **A** fossil block with six preserved turtle shells. Note that both plastron-up preserved shells have associated skull material. **B** zoomed in and rotated section, focusing on the position of the holotype skull and its associated shell. **C** interpretative line drawing of the holotype plastron. Note that scute sulci are drawn with a thick black line and labelled in bold font, and bone sutures are drawn in a thin black line and labelled in regular font. ab, abdominal; an, anal; eg, extragular; epi, epiplastron; ent, entoplastron; fem, femoral; gu, gular; hu, humeral; hyo, hyoplastraon; hyp, hypoplastron; ifm, inframarginal (roman numerals denote position); mar, marginal; mes, mesoplastron; pec, pectoral; per, peripheral; py, pygal; xi, xiphiplastron

Lyson et al. (2019) used the best-preserved cranium and lower jaw from the series of specimens found as the holotype for *Saxochelys gilberti* (DMNH EPV. 96000). The specimen was preserved upside down in association with six shells, an articulated hand, and other associated postcrania (Fig. 1). Two nearby shells are also preserved upside down and have associated cervical vertebrae suggesting either could belong with the holotype cranium. However, it was unclear which shell the holotype cranium and lower jaws was associated with at the time of publication of Lyson et al. (2019). Additional preparation on the block since Lyson et al. (2019) revealed the presence of a second skull associated with one of the plastron-up shells, strongly suggesting the second (Fig. 1B) plastron-up shell with associated cervical vertebrae belongs with the described holotype. We therefore regard this shell as part of the holotype and describe its morphology.

### CT scanning and digital segmentation

Since the initial description of *Saxochelys gilberti* by Lyson et al. (2019), we obtained a high-resolution X-ray micro-computed tomography (µCT) scan of the holotype skull of DMNH EPV. 96000. The skull fossil includes the cranium, mandible, partial hyoid apparatus, and first three cervical vertebrae. The fossil was scanned with a Bruker SkyScan2211 at the CT Core Facility of the Department of Geosciences at the University of Fribourg by Walter Joyce. The CT scan and relevant settings are deposited at the online repository MorphoSource (https://www.morphosource.org/projects/000456859), but key settings are also mentioned here: a voltage of 170 kV, a current of 115 µA, 140 ms exposure time and a 0.5 mm Copper filter were used during scanning. The CT slice data were segmented manually in Mimics v.24 (http://biomedical.materialise.com/mimics), and the resulting 3D models were exported as Polygon File Format (PLY) files. All 3D models are also deposited in MorphoSource, where they are linked to their parent scan data and housed in a single digital project that can be accessed under the following link: https://www.morphosource.org/projects/000456859. 3D models were rendered in Blender v2.71 (blender.org) for visualization purposes.

### Phylogenetic analysis

*Saxochelys gilberti* was originally included in a phylogenetic analysis by Lyson et al. (2019), who used a modified version of the Lyson et al. (2016) matrix, which itself is largely based on work by Lyson & Joyce (2011). This paracryptodiran matrix has recently been heavily modified by subsequent studies, particularly Joyce et al. (2020a), and Rollot et al. (2021a, 2022a), who modified and added a series of characters and changed a moderate amount of previous scorings based on new anatomical information gained through µCT-aided descriptions of a series of paracryptodires (e.g., Rollot et al., 2018; Evers et al., 2020, 2021; Pérez-García et al., 2021; Rollot et al., 2021a, 2022a, 2022b). In addition, several recent studies have used these matrices to add or modify specific taxa, such as *Denazinemys nodosa* in Spicher et al. (2023), who used the Rollot et al. (2022a) matrix as their scoring basis. Here, we use the Spicher et al. (2023) matrix for an updated phylogenetic assessment of *Saxochelys gilberti*. Based on our observations from digital dissections, we changed eight scorings: character 23 (0- > 1); 24 (0/1- > 0); 25 (1- > 0); 27 (1- > 0); 70(1- > 0); 72(0- > 1); 75(?- > 0); 76(?- > 1). The modified matrix is attached as Additional file 1. We performed a cladistic analysis in TNT v. 1.5 (Goloboff & Catalano, 2016; Goloboff et al., 2008). We ordered the same characters as in Spicher et al. (2023) (i.e., characters 3, 7, 11, 13, 15, 23, 24, 27, 30, 35–37, 42, 44, 56, 59, 76, 84, 91, 93, 94, 97) and used *Proganochelys quenstedtii* as the outgroup taxon. All characters were weighted equally, and we used the new technology search with all search algorithms enabled, we set the initial level of driven search to 30, and the number of times the minimum tree length should be obtained to 30. After initial analysis, we added a second round of TBR branch swapping. The resulting most parsimonious trees (MPTs) were summarized in a strict consensus tree.

### Systematic palaeontology

TESTUDINATA Klein, 1760 (Joyce et al., 2020b).

PARACRYPTODIRA Gaffney, 1975 (Joyce et al., 2021a).

BAENIDAE Cope, 1873 (Joyce et al., 2021a).

BAENODDA Brinkman, 2003 (Joyce & Lyson, 2015).

*SAXOCHELYS* Lyson et al., 2019

*SAXOCHELYS GILBERTI* Lyson et al., 2019

Type specimen: DMNH EPV.96000, an articulated skull with the mandible, hyoid apparatus (Lyson et al., 2019), three anterior cervical vertebrae, of which only the axis is well-preserved, and an associated, complete shell, preserved upside down so that currently only the plastron and carapacial margin are visible and prepared. Note that the three articulated cervical vertebrae and shell were not listed among the holotype material in the original description (Lyson et al., 2019). As the vertebrae are in the same fossil and articulated to the skull, they should be considered as part of the holotype. For the shell association, we provide a rationale in the methods section (above).

Holotype: see above.

Revised diagnosis: We herein provide a revised diagnosis for *Saxochelys gilberti*, reflecting our new anatomical observations. Observations from the newly prepared holotype shell of *Saxochelys gilberti* show that this specimen lacks the omega-shaped extragular-humeral sulcus that was listed as a diagnostic feature of the species by Lyson et al. (2019). In addition, Lyson et al. (2019) noted the absence of an omega-shaped femoral-anal sulcus as an additional diagnostic shell feature, but the holotype shell shows that this feature is also subject to intraspecific variation in *Saxochelys gilberti*. These new assessments highlight the strong levels of intraspecific variation that is present in shell scutes when a large sample of fossil individuals are available. Nevertheless, we observe the presence of five pairs of inframarginal scutes, which is unique among baenids and can be confirmed in the holotype as well as a well-preserved referred specimen that was figured by Lyson et al. (2019: Fig. 6). Additionally, the new cranial information we present reveals features that are herein interpreted as diagnostic of the species. *Saxochelys gilberti* can be identified as a baenid turtle based on several features that are usually found in combination in the crania of these turtles. These include an anteriorly placed entrance of the carotid arterial system along the parabasisphenoid-pterygoid suture, a long posterior process of the pterygoid that contacts the basioccipital and exoccipital, the presence of posteriorly broadening maxillary triturating surfaces, the presence of a pterygoid-postorbital contact (not present among all baenids, but generally only observed in paracryptodires and pleurodires among turtles) and the presence of reduced nasal bones. *Saxochelys gilberti* also shares with other baenids dorsally tall cervical vertebrae with a strong ventral keel and strong, medially positioned transverse processes that are associated with cervical ribs. Due to the high number of baenid taxa that have been described, it is difficult to assess specific morphological features that are unique in *Saxochelys gilberti*. Therefore, we here list a combination of features as diagnostic. None of these features may be unique to *Saxochelys gilberti*, but their combination, together with auxiliary information (e.g., stratigraphic and geographic), allow the identification of *Saxochelys gilberti* material*. Saxochelys gilberti* has a jugal that is excluded from the orbital margin by a posterodorsal process of the maxilla that contacts the postorbital, a quadrate-supraoccipital contact, a pterygoid-postorbital contact, an occipital condyle that is exclusively formed by the basioccipital, a foramen orbito-nasale that is exclusively formed by the palatine and prefrontal without a maxilla contribution, the absence of a nasal-maxilla contact and presence of a small narial notch between both bones, an oblique ridge along the posteromedial margin of the pterygoid that parallels the basioccipital suture, the presence of sharp ridges that trend along the anterior surface of the parabasisphenoid from the clinoid processes to the sella turcica, a sulcus for the hyomandibular nerve in the prootic and an associated fossa for the geniculate ganglion that lies medial to the canalis cavernosus and, thus, fully within the prootic, and a rod-like epipterygoid that is excluded from the trigeminal foramen but parallels its anteroventral margin alongside the descending parietal process. In addition, *Saxochelys gilberti* shows imprinted scute sulci alongside a weakly textured temporal bone surface. The mandible of *Saxochelys gilberti* has a well-developed processus dentofacialis, reduced sizes of the foramen auriculotemporalis and foramen dentofaciale-major, and a symphyseal tongue shelf.

Type locality and horizon: DMNH Locality 6302 (Turtle Ridge locality), northwest of Marmarth, Bucklin Township, Slope County, North Dakota; Hell Creek Formation, latest Maastrichtian, Late Cretaceous (Lyson et al., 2019).

Referred material: As in (Lyson et al., 2019).

## Results

### Description

*Holotype shell.* The shell that we newly recognize as belonging to the holotype skull is well preserved (Fig. 1). The straight carapace length is about 29 cm and the length of the plastron is precisely 259,5 mm. The shell is oblong in shape and the posterior edge of the carapace is weakly scalloped to a pygal notch. The shell has a distinct crenulated texture. The anterior and posterior lobes of the plastron are approximately subequal in size. The anterior lobe is broadly rounded and the posterior lobe is shaped like an inverted trapezoid. Both the sutures and the sulci are clearly visible (Fig. 1C). The epiplastra are crescent shaped and the entoplastron is rhomboidal. The hyo- and hypoplastra form robust axillary and inguinal buttresses, respectively. The mesoplastra narrow medially and broadly contact each other along the midline.

The paired gular scutes are small and largely restricted to the epiplastra, but overlap the anterior portion of the entoplastron centrally. The extragulars are larger than the gulars, but lack the distinctive sigmoid-shaped contact with the humerals posteriorly as found in several of the shells described by Lyson et al. (2019). The humeral scutes cover most of the anterior plastral lobe and have a slightly curved, anteriorly concave contact with the posteriorly adjacent pectorals (Lyson et al., 2019). Laterally, the pectoral scutes contact inframarginals scutes I–III laterally and the abdominal scutes contact inframarginals II–V. *Saxochelys gilberti* is highly unusual for baenid turtles in having five pairs inframarginals instead of the regular four pairs (Joyce & Lyson, 2015). Lyson et al. (2019) originally only reported and indicated four pairs inframarginals for *Saxochelys gilberti*. However, the specimen DMNH EPV.98812 shown in their Fig. 6 already suggests the presence of an additional pair of inframarginals anterior to those labelled in the figure. The supernumerary inframarginal pair is partially covered by matrix in the anterior bridge region, possibly explaining why only four pairs of inframarginals were recognized originally. In other baenids, the fourth inframarginal usually extends across the mesoplastral-hypoplastral suture and extends into the inguinal buttress (Joyce & Lyson, 2015). In both *Saxochelys gilberti* specimens that show the posterior inframarginals, it is also the fourth inframarginal that extends over the mesoplastral-hypoplastral suture. However, the fourth inframarginal does not extend into the inguinal buttress. Thus, the supernumerary inframarginal of *Saxochelys gilberti* is either caused by a division of the fourth inframarginal of other baenids, or by the addition of a scute posterior to the fourth inframarginal. The pectorals are larger than the abdominals, and both pectorals and abdominals reach into the auxillary and inguinal notches, respectively. The midline sulcus between right and left plastral scutes is sigmoidally-curved in the pectoral-abdominal section, but less irregular on the anterior and posterior plastral lobes. The femoral scutes have a sigmoidal contact with the anal scutes, resulting in an omega-shaped suture when both sides are viewed together. This constitutes a distinct difference from the straight contact found in other *Saxochelys gilberti* shells (Lyson et al., 2019). The prepared holotype shell of *Saxochelys gilberti* shows that the bridge extends up to the seventh peripheral posteriorly. Anteriorly, the bridge extends at least until the second peripheral, but a contact with the first peripheral cannot be excluded. As already reported for the species (Lyson et al., 2019) the holotype confirms the presence of eleven peripherals and twelve marginal scutes.

**Fig. 2:**
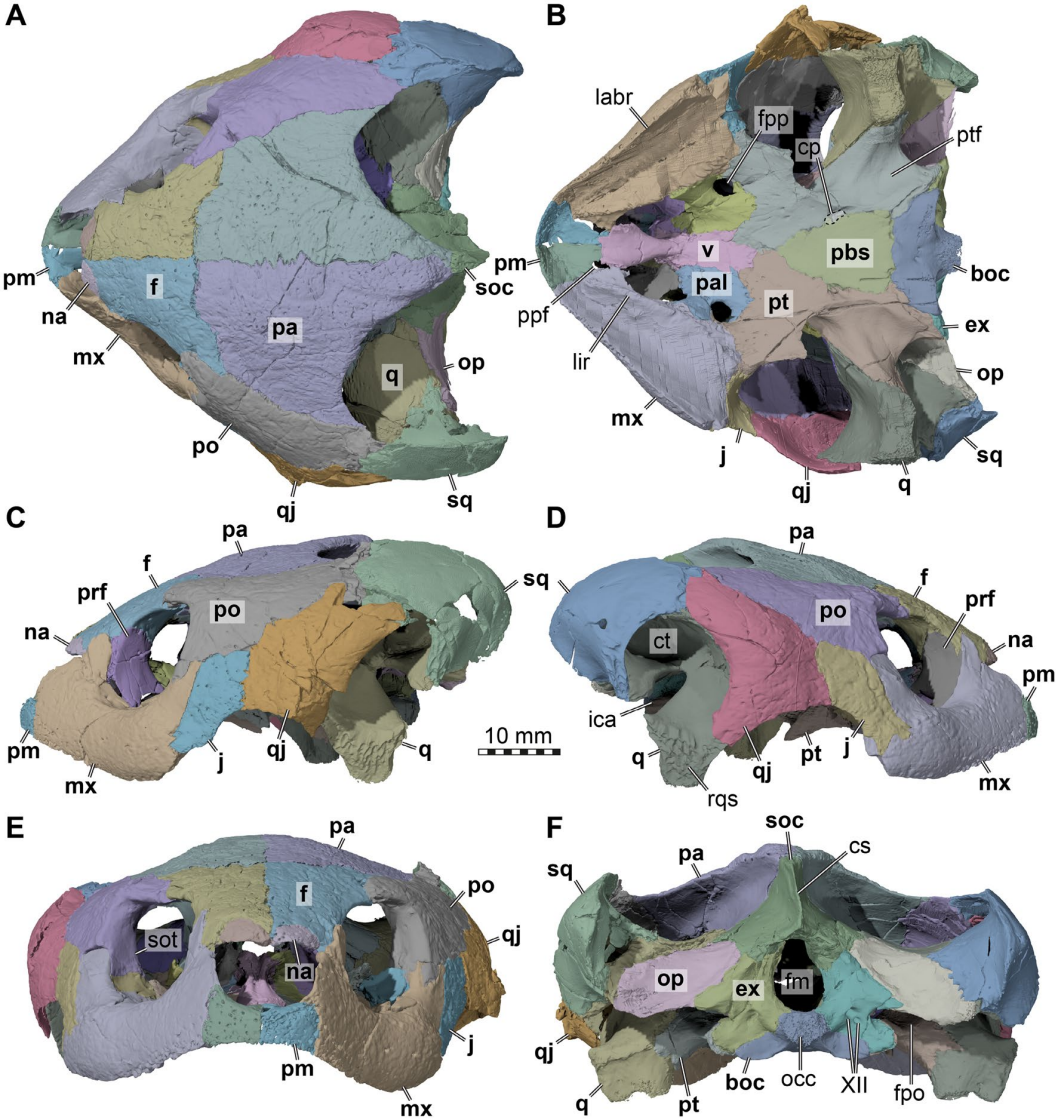
3D renderings of the cranium of the holotype of *Saxochelys gilberti* (DMNH EPV.96000). **A** dorsal view. **B** ventral view. **C** left lateral view. **D** right lateral view. **E** anterior view. **F** posterior view. Note that bones are labelled in bold font, and traits are labelled in regular font. boc, basioccipital; cp, carotid pit; cs, crista supraoccipitalis; ct, cavum tympani; ex, exoccipital; f, frontal; fm, foramen magnum; fpp, foramen palatinum posterius; fpo, fenestra postotica; ica, incisura columellae auris; j, jugal; labr, labial ridge; lir, lingual ridge; mx, maxilla; na, nasal, occ, occipital condyle; op, opisthotic; pa, parietal; pal, palatine; pbs, parabasisphenoid; pm, premaxilla; po, postorbital; ppf, foramen praepalatinum; prf, prefrontal; pt, pterygoid; ptf, pterygoid fossa; q, quadrate, qj, quadratojugal; rqs, rough quadrate surface; soc, supraoccipital; sot, septum orbito-temporale; sq, squamosal; v, vomer; XII, foramina for the hypoglossal (CN XII) nerve

*General features of the skull.* The skull of DMNH EPV.96000 is well preserved (Fig. 2). The skull is wedge-shaped, relatively broad, moderately high, and moderately short. The cheek and temporal emarginations are moderately deep. The orbits are largely laterally-oriented, although parts of the orbital fossa can be seen in dorsal view. The temporal bones have surface texture consisting of numerous small pits and grooves embedded in an otherwise relatively smooth surface. Some cranial sulci are visible across the skull roof. The snout is anteriorly protruding, so that the external naris is anterodorsally-oriented. The supraoccipital crest is short. The triturating surfaces are relatively broad and the mandible robust.

**Fig. 3:**
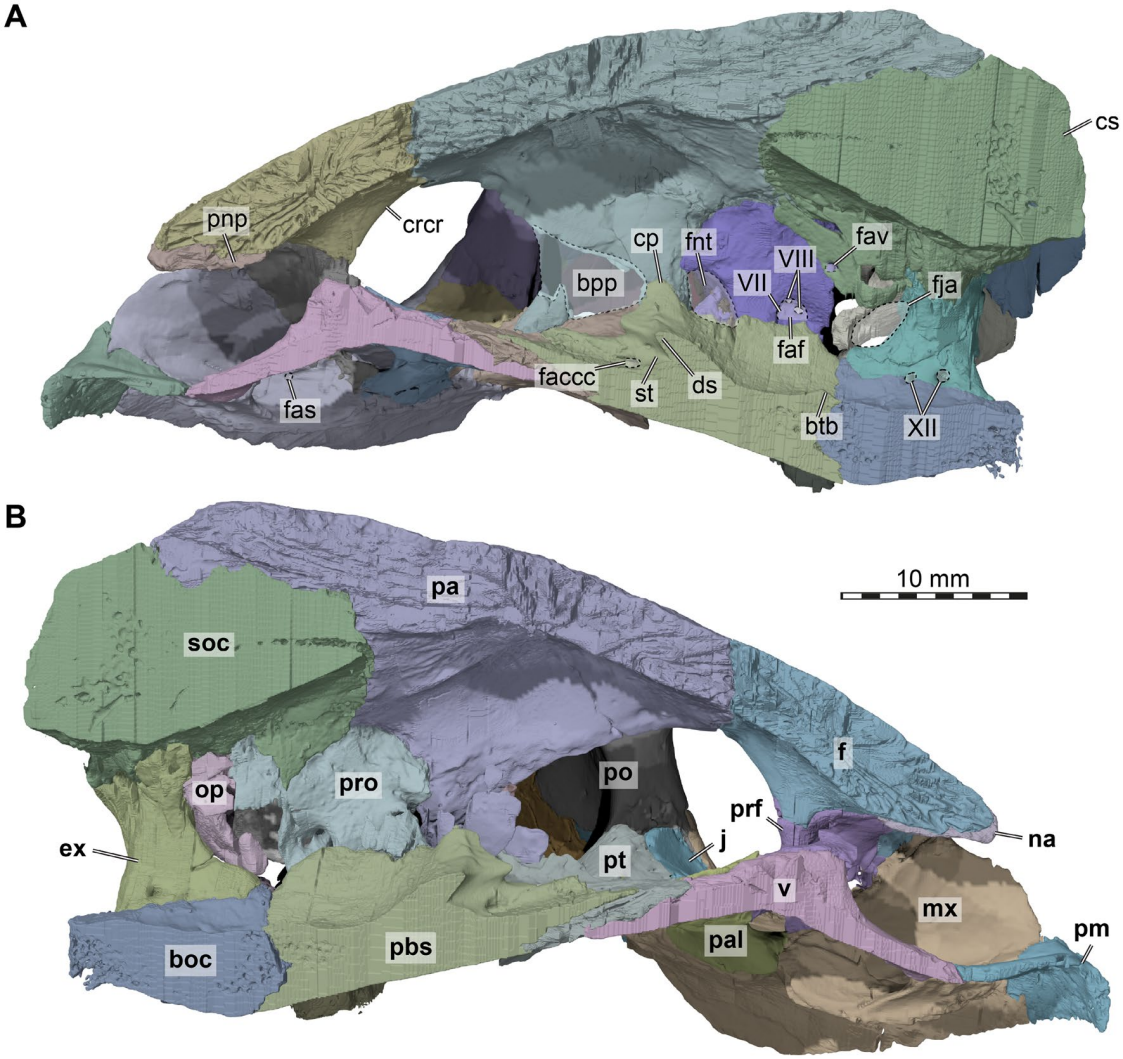
3D renderings of the internal cranium of the holotype of *Saxochelys gilberti* (DMNH EPV.96000). **A** internal (medial) view of right side of cranium. **B** internal (medial) view of left side of cranium. Note that median bones have been sectioned in the midline to produce these images. Also note that bones are labelled in bold font, and traits are labelled in regular font. boc, basioccipital; bpp, broken part of parietal; btb, basis tuberculi basalis; cp, clinoid process; crcr, crista cranii; cs, crista supraoccipitalis; ds, dorsum sellae; ex, exoccipital; f, frontal; faccc, foramen anterius canalis carotici cerebralis; faf, fossa acustico-facialis; fas, foramen alveolare superius; fav, foramen aquaducti verstibuli; fja, foramen jugulare anterius; fnt, foramen nervi trigemini; j, jugal; mx, maxilla; na, nasal; op, opisthotic; pa, parietal; pal, palatine; pbs, parabasisphenoid; pnp, posterior nasal process; pm, premaxilla; po, postorbital; prf, prefrontal; pt, pterygoid; soc, supraoccipital; st, sella turcica; v, vomer; VII, foramen for the facial (CN VII) nerve; VIII, foramina for the acoustic (CN VIII) nerve; XII, foramina for the hypoglossal (CN XII) nerve

*Nasal*. Both nasals are preserved in DMNH EPV.96000 (Figs. 2A, C, D, E, 3). The nasal is a small element at the anterior end of the skull roof, where it forms the anterodorsal margin of the external naris and parts of the roof of the fossa nasalis. The nasal is much smaller than in most other baenodds (e.g., Joyce & Lyson, 2015; Rollot et al., 2018; Spicher et al., 2023.) Externally, the bone seems to only contact the frontal, and has only a small point of contact with its counterpart, so that the nasals seem nearly separated (Fig. 2A, E). This separation of the nasals along the midline can indeed be found in some baenids, such as *Palatobaena cohen* (Joyce & Lyson, 2015). However, the nasal of DMNH EPV.96000 extends posteroventrally underneath the frontal (Fig. 3A, B), where it has a long midline contact with the corresponding nasal. Within the fossa nasalis, the nasal contacts the prefrontal posterolaterally (Fig. 3B), a contact that is absent on the external skull surface. Thus, although the external exposure of the nasal is mediolaterally broader than anteroposteriorly long, this is reversed when the nasal is viewed in isolation. The posterior contact of the nasal with the frontal is curved in a way that the frontal frames the nasal both medially and laterally, resulting in the small external midline contact of the nasals mentioned above (Fig. 2A, E). Laterally, there is a notch between the nasal and maxilla which prevents a contact of these two bones (Fig. 2C, D), as has been reported for *Chisternon undatum* (Gaffney, 1982). The preserved margin of the maxilla in DMNH EPV.96000 indicates that this posterodorsal notch of the margin of the external naris is not an artifact but original morphology. Our novel observations contradict the original assessment of Lyson et al. (2019), in which the nasal was originally reported to contact the maxilla.

*Prefrontal*. Both prefrontals are reasonably well-preserved in DMNH EPV.96000 (Figs. 2C, D, 3), although the articulation with the vomer and palatines is affected by breakage that makes it difficult to ascertain the details of the area in which these bones contact one another. The prefrontal is reduced in size to a simple vertical sheet of bone in the anterior wall of the orbit, and lacks a dorsal horizontal sheet that takes part in the dorsal skull roof (Fig. 2A, C, D). It is positioned in the anterior part of the skull but completely reduced from contributing to the dorsal skull roof (Fig. 2A). The prefrontal of DMNH EPV.96000 has only a very minor contribution to the orbital margin, and is medially retracted into the orbital fossa itself (Fig. 2C, D). The prefrontal contacts the frontal dorsally and the maxilla laterally. Moreover, the dorsal process also slightly contacts the nasal anteromedially within the nasal cavity (Fig. 3B). The ventral part of the prefrontal bifurcates into two short processes that frame the dorsal margin of the foramen orbito-nasale. The ventral margin of the foramen orbito-nasale is formed by the palatine, without contributions of the maxilla, as in many other younger, more derived baenids (e.g., *Eubaena cephalica*: Rollot et al., 2018; *Denazinemys nodosa*: Spicher et al., 2023), but contrary to early baenids (e.g., *Arundelemys dardeni*: Evers et al., 2021) or pleurosternids (e.g., *Pleurosternon bullockii*: Evers et al., 2020). The medial process of the prefrontal of DMNH EPV.96000 contacts the palatine and the vomer.

*Frontal*. Completely preserved in DMNH EPV.96000, the frontals are paired bones that are exposed on the anterior region of the skull roof that contribute to the anterior half of the dorsal orbital margin (Fig. 2A). The frontals are flat, and they are wider posteriorly than anteriorly. The frontals of DMNH EPV.96000 are mediolaterally broad, forming a wide interorbital bar that contributes to the overall broad yet short skull morphology (Fig. 2A). The frontals contact one another along the midline. The bone also contacts the parietal posteriorly along its entire width and the postorbital posterolaterally (Fig. 2A). The contact between the frontals and the parietals is nearly straight, as in other eubaenines (e.g., *Denazinemys nodosa*: Spicher et al., 2023; *Stygiochelys estesi*: Gaffney & Hiatt, 1971; *Baena arenosa*: Gaffney, 1972a). In dorsal view of DMNH EPV.96000, the frontal extends in between the parietal-postorbital contact for a short distance with a tapering posterodorsal process. Anterolaterally, the frontal contacts the maxilla (Fig. 2C, D), and the frontal covers the dorsal process of the prefrontal along its anterior end. The frontal contacts the nasal by overlapping it along its anterior margin. Hereby, right and left frontals form a short anteriorly directed midline process that nearly separates the nasals entirely in dorsal view (Figs. 2A, E, 3A, B). On the ventral surface, the frontal bears only a low and short crista cranii (Fig. 2A). Both these ridges are widely spaced, resulting in a broad and weakly defined sulcus olfactorius.

**Fig. 4:**
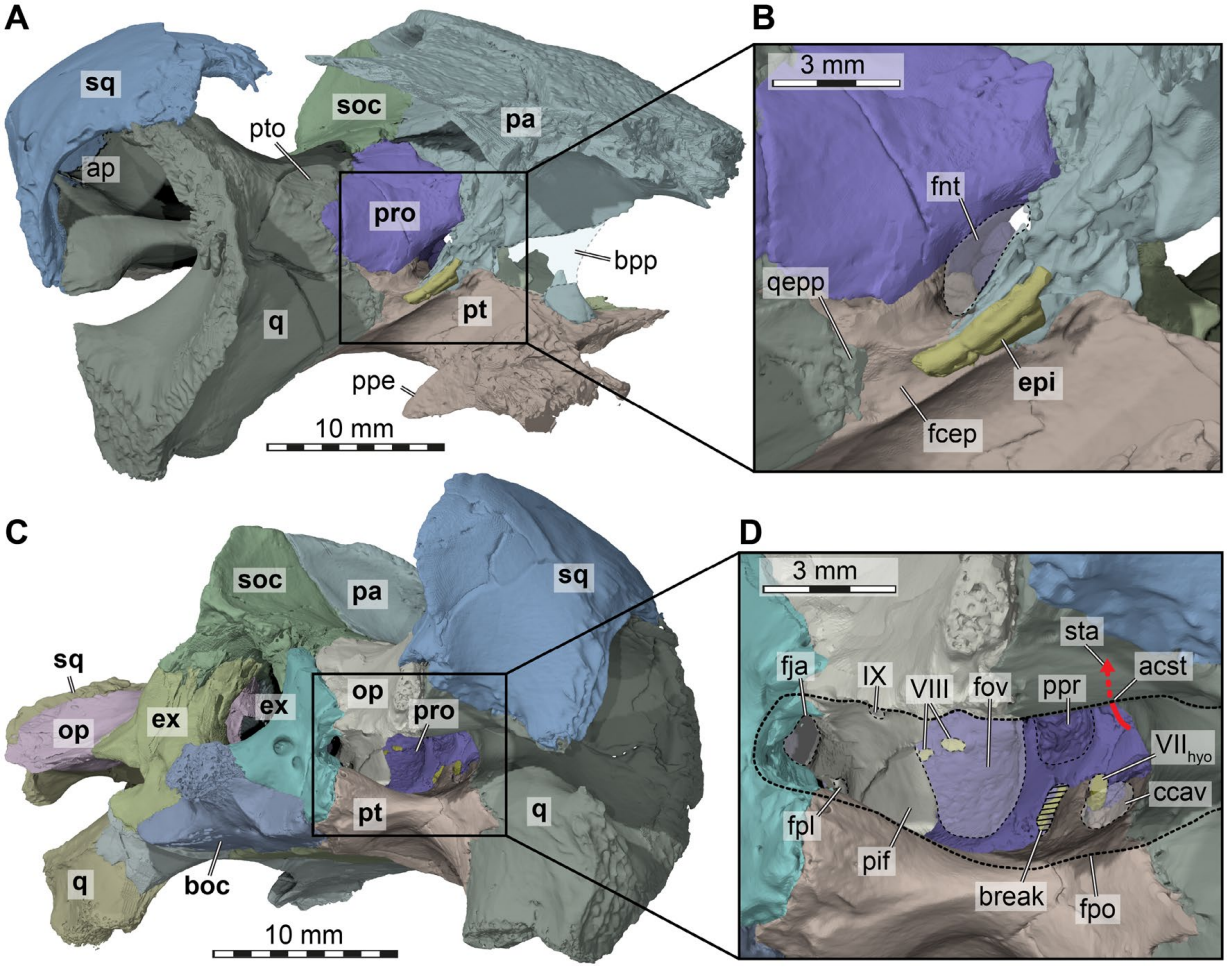
3D renderings of the cranium of the holotype of *Saxochelys gilberti* (DMNH EPV.96000). **A** anterolateral view into the partial right cranium. **B** close-up of trigeminal region. **C** right posterolateral view into the cranium. **D** close-up of fenestra postotica and internal features of cavum acustico-jugulare. Note that bones are labelled in bold font, and traits are labelled in regular font. acst, aditus canalis stapedio temporalis; ap, antrum postoticum; boc, basioccipital; bpp, broken part of parietal; ccav, canalis cavernosus (posterior opening); epi, epipterygoid; ex, exoccipital; fcep, fossa cartilaginis epipterygoidei; fja, foramen jugulare anterius; fnt, foramen nervi trigemini; fov, fenestra ovalis; fpl, fenestra perilymphatica; fpo, fenestra postotica; IX, external foramen for the glossopharyngeal (CN IX) nerve; op, opisthotic; pa, parietal; pif, processus interfenestralis; ppe, processus pterygoideus externus (transverse process of pterygoid); ppr, posterior prootic recess; pro, prootic; pt, pterygoid; pto, processus trochlearis oticum; q, quadrate; qepp, quadrate epipterygoid process; soc, supraoccipital; sq, squamosal; sta, stapedial artery; v, vomer; VII_hyo_, groove for the hyomandibular ramus of the facial (CN VII) nerve; VIII, foramina for the acoustic (CN VIII) nerve

*Parietal*. The parietals of DMNH EPV.96000 are nearly completely preserved and fully articulated (Figs. 2A, C, D, 3, 4A). However, the ventral ends of the descending processes are partially broken along the contact with the palate and basicranium on both sides, making it difficult to fully reconstruct the parietal morphology in this region of the skull. The parietal contacts the frontal, postorbital, supraoccipital, the other parietal, prootic, pterygoid, and epipterygoid, and these contacts are detailed further below.

The posterior margin of the parietal is completely intact, showing that the upper temporal emargination is moderately deep: although the foramen stapedio-temporale is covered in dorsal view, the margin of the emargination is deeply concave and exposes much of the upper temporal fossa (Fig. 2A). This differs from the deeper emarginations of some other eubaenines (e.g., *Eubaena cephalica*: Rollot et al., 2018; *Denazinemys nodosa*: Spicher et al., 2023; *Stygiochelys estesi*: Gaffney & Hiatt, 1971) and early baenids such as *Lakotemys australodakotensis* (Rollot et al., 2022b) or *Trinitichelys hiatti* (Rollot et al., 2022a), but is similar to the potentially closely related *Baena arenosa* and *Chisternon undatum* (Gaffney, 1972a).

The parietal of DMNH EPV.96000 is a large bone in the posterior and central parts of the skull, which forms large parts of the skull roof with a dorsal plate that is horizontally-oriented (Fig. 2A). In addition, the parietal has adescending process that diverges vertically ventrally from the dorsal plate and forms parts of the lateral wall of the braincase (Figs. 3, 4A). The dorsal plate of the parietal forms the largest part of the upper temporal emargination, where it contacts the postorbital. However, a contact with the squamosal along its posterior margin is absent. Posteriorly, the parietal forms a midline process that overlies the supraoccipital. Laterally, the parietal has a long contact with the postorbital, and meets the frontal anteriorly. Along the midline, the parietal forms an interdigitated contact with its contralateral neighbour (Fig. 2A). The parietal is extremely thick along the skull roof, and is dorsally slightly textured, although weak scute sulci are also preserved (see below).

The descending process of the parietal overlaps the supraoccipital posteriorly. Ventrally along the medial end of the otic capsule, it is tightly sutured to the prootic. The parietal forms the dorsal and anterodorsal margin of the trigeminal foramen (Fig. 4A, B), which is otherwise posteriorly framed by the prootic and ventrally bound by the pterygoid. A posteroventral process that would exclude the prootic from the margin of the trigeminal foramen, as is sometimes found (e.g., *Denazinemys nodosa*: Spicher et al., 2023), is absent in DMNH EPV.96000. The trigeminal foramen of DMNH EPV.96000 is relatively small and narrow, although breakage in this region obscures the original shape of the foramen on the left side. A contact of the parietal with the pterygoid is present anterior to the trigeminal foramen (Figs. 3A, 4A). A dorsal suture on the pterygoid, as well as a broken but articulated piece of the right parietal, indicates that the parietal of DMNH EPV.96000 had an elongated contact extending over nearly the entire pterygoid surface (Figs. 3A, 4A). However, as preserved, most of this original contact is broken, making the lateral wall of the braincase appear anteriorly shorter than it would have been during life. However, the preserved morphology indicates that the parietal did not extend all the way to contact the palatine. A last contact of the parietal is with the epipterygoid, which lies against the lateral surface of the descending process of the parietal anterior to the position of the trigeminal foramen (Fig. 4A).

*Postorbital*. Both postorbitals of DMNH EPV.96000 are well-preserved despite some small damage on the posterior edge of the left postorbital (Fig. 2A, C, D, E). The postorbital is a flat and elongate bone that is exposed on the lateral region of the posterior half of the skull roof. The bone also covers a large anterior part of the temporal fossa and forms a small part of the margin of the upper temporal emargination, preventing a parietal-squamosal contact (Fig. 2A). A relatively broad contact with the squamosal is visible posteriorly. The postorbital forms two main processes anteriorly, which frame the posterior margin of the orbit. The dorsal process contacts the frontal anteromedially and the ventral process extends toward the jugal and maxilla, hereby forming the posterior margin of the orbit (Fig. 2C, D, E). The postorbital-jugal contact area extends medially into the orbit, forming a septum orbito-temporale as recently described for several baenids (Fig. 2E; e.g., Evers et al., 2021; Rollot et al., 2022a, 2022b). Lyson et al. (2019) reported a contact between the postorbital and the maxilla, which is commonly observed in baenids, including early taxa such as *Arundelemys dardeni* (Evers et al., 2021), *Lakotemys australodakotensis* (Rollot et al., 2022b), and *Trinitichelys hiatti* (Rollot et al., 2022a), but also commonly seen in eubaenines such as *Boremys pulchra* (Brinkman & Nicholls, 1991), *Eubaena cephalica* (Gaffney, 1972a; Rollot et al., 2018), and *Denazinemys nodosa* (Spicher et al., 2023). A postorbital-maxilla contact, however, is absent in most turtles generally, including outgroups such as pleurosternids (Evers et al., 2020), and also in eubaenines that have been proposed to be closely related to *Saxochelys gilberti*, particularly *Baena arenosa* and *Chisternon undatum* (Gaffney, 1972a). Here, we confirm that the contact is present in DMNH EPV.96000 (Fig. 2A, C, D, E), as observed by Lyson et al. (2019). Along the base of the septum orbito-temporale of DMNH EPV.96000, there is a short contact of the postorbital with the pterygoid, which is an unusual contact for turtles in general, but which appears in multiple baenids in which this area is well-preserved (e.g., *Arundelemys dardeni*: Evers et al., 2021; *Denazinemys nodosa*: Spicher et al., 2023). This contact is best appreciated on the right side of DMNH EPV.96000, as the pterygoid is slightly disarticulated on the left side. The postorbital also contacts the quadratojugal posteroventrally (Fig. 2C, D).

*Jugal*. The jugals are well-preserved in DMNH EPV.96000 (Fig. 2C, D, E). The jugal is exposed in the center of the lateral region of the skull. The jugal contacts the quadratojugal with its posterior margin, contributes to the anterior half of the lower temporal emargination and contacts the postorbital dorsally. Along this contact, it forms the septum orbito-temporale along its medial process (Fig. 2E), which also contacts the pterygoid medially. A contact with the palatine is absent. The jugal of DMNH EPV.96000 does not contribute to the margin of the orbit anteriorly (Fig. 2A, C, D, E), which differs from *Baena arenosa* and *Chisternon undatum* (Gaffney, 1972a) but is similar to some other baenodds with completely reduced orbit contributions of the jugal (see postorbital). However, the jugal of DMNH EPV.96000 is nevertheless expressed in the orbital fossa, just medial to the orbital margin (Fig. 2E). The jugal of DMNH EPV.96000 broadly contacts the maxilla ventrally, but the jugal is dorsally positioned with respect to the level of the maxillary lingual ridge.

*Quadratojugal*. The quadratojugals of DMNH EPV.96000 are both preserved (Fig. 2C, D), despite some crushing on the left bone ventrally and the right bone dorsally. A small part is also missing on the left quadratojugal posteriorly near the contacts with the postorbital and the squamosal. The quadratojugal forms large parts of the posterior lateral region of the skull, and contacts the squamosal posterodorsally, the postorbital anterodorsally, the quadrate posteromedially, and the jugal anteriorly (Fig. 2C, D). The quadratojugal frames the lateral margin of the quadrate, hereby forming the entire anterior margin of the cavum tympani (Fig. 2C, D). Posteroventrally, the quadratojugal extends along the quadrate without reaching the area of the mandibular articulation (Fig. 2C, D). However, on both sides, it seems possible that the quadratojugal once extended farther ventrally down the quadrate. This is difficult to discern, as the quadrate is roughened laterally in this area (Fig. 2D), which could be an indication for an ongoing quadratojugal articulation, but it could also simply indicate insertion of soft tissues related to the tympanic membrane. The quadratojugal forms the posterior half of the lower temporal emargination (Fig. 2C, D).

*Squamosal*. Both squamosals are preserved and articulated in DMNH EPV.96000 (Figs. 2A, C, D, F, 4), despite some breaks and missing portions on the medial edge of the left squamosal. The bone is exposed on the most posterior region of the skull and contributes to the skull roof, where it contacts the postorbital and quadratojugal, but not the parietal (Fig. 2A). The squamosal also broadly contributes to the upper temporal emargination medially. Within the temporal fossa, the squamosal broadly contacts the quadrate (Fig. 2A) and forms a bony cap over the antrum postoticum (Fig. 4A). Hereby, it forms the posterodorsal margin of the cavum tympani (Figs. 2C, D, 4A). Posteriorly, the squamosal forms a thin, vertical edge that is continuous anteromedially with the margin of the upper temporal emargination (Figs. 2F, 4B). On the posterior side of the skull, the squamosal also contacts the opisthotic posteromedially (Fig. 2F).

*Premaxilla*. The premaxillae of DMNH EPV.96000 are well-preserved (Figs. 2B, E, 3). The bone is positioned at the most anterior region of the skull and forms the anterior part of the rostrum, where it forms the ventral margin of the external naris and parts of the labial ridge that is laterally confluent with the maxilla. It contacts the maxilla laterally, the vomer posteriorly, and its counterpart along a long median suture (Fig. 2B). The premaxilla is longer than wide and floors much of the nasal cavity. At the posterior end of the posterior process, the premaxilla frames the foramen praepalatinum with the laterally adjacent maxilla and the posteriorly adjacent vomer (Fig. 2B). Although this foramen is damaged on both skull sides, it is clearly visible on the right side. The premaxilla also narrowly participates in forming the triturating surface and the anterior part of the labial ridge. The posterior processes of the premaxilla form a weak tongue groove together with the short lingual ridge of the maxilla (Fig. 2B).

*Maxilla*. Both maxillae are completely preserved (Figs. 2B, C, D, E, 3). The maxilla forms a broad part of the snout and is, therefore, located at the anterior region of the skull. The maxilla forms the anterior and ventral margin of the orbit, the major part of the labial margin and even a small part of the posterior margin. The bone contacts the jugal and postorbital posteriorly, the pterygoid posteromedially, the palatine posteromedially, the premaxilla anteriorly, the prefrontal medially along the ascending process, and the frontal dorsally. Lyson et al. (2019) described a contact of the ascending process of the maxilla with the nasal bone, but the 3D models of DMNH EPV.96000 suggest that this contact was absent and instead these bones are separated by a small notch (see nasal; Fig. 2C, D, E). The ascending process of the maxilla forms the lateral wall of the fossa nasalis. Within the fossa nasalis, the maxilla contacts the vomer anteromedially, and this is also seen in ventral view (Fig. 2B). The medial process of the maxilla underlies the jugal and floors the lateral half of the orbital cavity. At its posterior end, the maxilla has a dorsally-upcurved process within the orbital margin that contacts the postorbital. This process of the maxilla is a relatively thin lappet of bone that also overlaps the jugal in this area. The superficial contact of the maxilla and postorbital cause the jugal to be excluded from the orbital margin, as already described by Lyson et al. (2019). Within the floor of the orbital cavity are two principal openings into the canalis alveolare superius, which is the main internal maxillary canal, from which several neurovascular foramina diverge into the labial region of the maxilla. The smaller one is near the anterior contact with the jugal, and likely the foramen supramaxillare. Another larger foramen also leads into the maxilla from within the orbital cavity floor. This second foramen is more anterodorsally-positioned, at the base of the posterior surface of the ascending process of the maxilla. The foramen alveolare superius is also visible on the medial surface of the maxilla within the fossa nasalis (Fig. 3A). The maxillary triturating surfaces are narrow anteriorly at the contact with the premaxillae and broaden posteriorly (Fig. 3B), as is common in baenids. The labial ridge is deep and sharp-edged. The lingual ridge is present but not continuous along the entire lingual margin. Instead, the ridge is prominent anteriorly, where it forms a ventrally concave protuberance, which lines the tongue groove. Posteriorly, the lingual ridge fades (Fig. 3B).

*Vomer*. The vomer is entirely preserved (Figs. 2B, 3). It is a single narrow and elongated bone in the skull midline. The vomer consists of a plate-like posterior part that is integrated into the palate by being laterally framed by the palatines and by contacting the pterygoids posteriorly. An anteroventral process of the vomer contacts the premaxillae and maxillae, and short dorsal processes contact the prefrontals and frame the sulcus vomeri. Dorsally, this plate-like part bears no trough posterior to the sulcus vomeri, and the ventral surface is also smooth, so that a ventral keel is absent (Fig. 2B). Anterodorsally, the vomerine process that contacts the prefrontal is extremely low (Fig. 3), also resulting in a low sulcus vomeri. The anteroventral process of the vomer also bears no ventral ridge, and contributes to the foramina praepalatina anteriorly (see premaxilla; Fig. 3B).

*Palatine*. Both palatines of DMNH EPV.96000 are well-preserved (Fig. 2B). The palatine is a plate-like bone with a constricted lateral process that extends between the foramen orbito-nasale and the foramen palatinum posterius (Fig. 2B). Laterally to these foramina, the lateral process expands anteroposteriorly and contacts the maxilla along the lingual margin of that bone. However, the palatine does not contribute to the triturating surface (Fig. 2B). The foramen palatinum posterius is mostly framed by the palatine, but the pterygoid contributes to the posterior margin (Fig. 2B). The palatine forms the entire posterior margin of the foramen orbito-nasale, which is otherwise only framed by the prefrontal, without maxilla or vomer contributions. This observation contradicts those made by Lyson et al. (2019), who described the maxilla as forming the anterolateral third of the foramen orbito-nasale. A midline contact between the palatines is prohibited by the vomer (Fig. 2B). Posteriorly, the palatine contacts the pterygoid only and a contact with the jugal, as originally described by Lyson et al. (2019), is clearly absent (Fig. 2B).

*Quadrate*. The quadrates are well-preserved in DMNH EPV.96000 (Figs. 2B, C, D, F, 3, 4A), and only a small piece of the left quadrate is broken toward the anterior margin of the cavum tympani. The quadrate is positioned posteriorly in the skull and forms a major part of the middle ear and floors the temporal fossa dorsally. It forms the cavum tympani and the mandibular condyle. The bone contacts the squamosal posteriorly, the supraoccipital medially, the prootic anteromedially, the quadratojugal anterolaterally, the opisthotic posteriorly, and the pterygoid medially. A contact with the epipterygoid is not preserved (Fig. 4A), but may have been present depending on whether the epipterygoid of DMNH EPV.96000 is fully ossified posteriorly (see epipterygoid).

The quadrate is medially removed from the lateral side of the skull, as it is anteriorly overlapped by the quadratojugal and posteriorly by the squamosal (Fig. 2C, D). Thus, the quadrate is largely excluded from the margin of the cavum tympani, with the possible exception ventrally, along the lateral side of the articular process of the quadrate. The lateral surface of this process is covered in deep irregular ridges and troughs (Figs. 2D, 4A). These continue along the anterolateral margin of the quadrate towards the quadratojugal. Thus, there is a possibility that this irregular surface texture indicates a further extent of the quadratojugal than is preserved. Alternatively, these ridges could indicate the former presence of soft tissues related to the articulation of the tympanum. Muscle insertions are unlikely as an explanation for the roughened texture, as mandibular muscles in turtles do not seem to attach to the lateral surface of the articular process of the quadrate (Evers et al., 2022; Werneburg, 2015). Posterodorsal to the articular process, the incisura columella auris forms a broadly open notch in the quadrate (Figs. 2C, D, 4A, B).

Together with the prootic, the quadrate forms the process trochlearis oticum, which is formed as an inconspicuous structure along the anterodorsal surface of the otic capsule (Fig. 4A). Between the prootic and quadrate, the canalis stapedio-temporalis ascends vertically from within the cavum acustico-jugulare, and opens dorsally in the foramen stapedio-temporale. Within the floor of the temporal fossa, the quadrate has a medial process, which superficially extends between the prootic and opisthotic, and contacts the supraoccipital (Fig. 2A). This contact is unusual in turtles (e.g., Gaffney, 1979a), and also absent in early baenids such as *Arundelemys dardeni* (Evers et al., 2021), and *Lakotemys australodakotensis* (Rollot et al., 2022b), although it may be present in *Trinitichelys hiatti* (Rollot et al., 2022a). This contact seems, however, regularly present in baenodds (e.g., *Eubaena cephalica*: Rollot et al., 2018; *Denazinemys nodosa*: Spicher et al., 2023; *Stygiochelys estesi*: Gaffney & Hiatt, 1971; *Plesiobaena antiqua*: Brinkman, 2003; *Palatobaena cohen*: Lyson & Joyce, 2009a).

On the anterior surface of the quadrate, the epipterygoid process of DMNH EPV.96000 is incompletely preserved on both sides (Fig. 4A), although it seems more complete on the left side. Thus, it is not entirely clear if a quadrate-epipterygoid contact may have existed, or if the gap between both bones was filled with cartilage instead.

*Epipterygoid*. Both epipterygoids are preserved in DMNH EPV.96000, despite the presence of breaks and crushing on both sides of the skull in the area of the lateral braincase wall. Although the left epipterygoid is more completely preserved than the right, the trigeminal area is better preserved on the right side (Fig. 4A, B). The epipterygoid is a small, elongated and rod-like bone (Fig. 4B) that is morphologically very similar to the epipterygoid described for *Plesiobaena antiqua* (Smith et al., 2023). The epipterygoid of DMNH EPV.96000 is located just anteroventral to the trigeminal foramen without contributing to its margin (Fig. 4B), again as in *Plesiobaena antiqua* (Smith et al., 2023). The epipterygoid of DMNH EPV.96000 contacts the pterygoid medially on its posterior half and the parietal dorsally and medially. A contact with the quadrate cannot be excluded but is presently not preserved. Instead, there is a groove posteroventral to the posterior end of the epipterygoid, the fossa cartilaginis epipterygoidei, which extends toward the epipterygoid process of the quadrate (Fig. 4B). This fossa may have been filled with cartilage, establishing a cartilaginous bridge between the epipterygoid and quadrate. Lyson et al. (2019) observed this groove and interpreted it as a facet for the (lost) epipterygoid, and did not observe the bone itself.

**Fig. 5:**
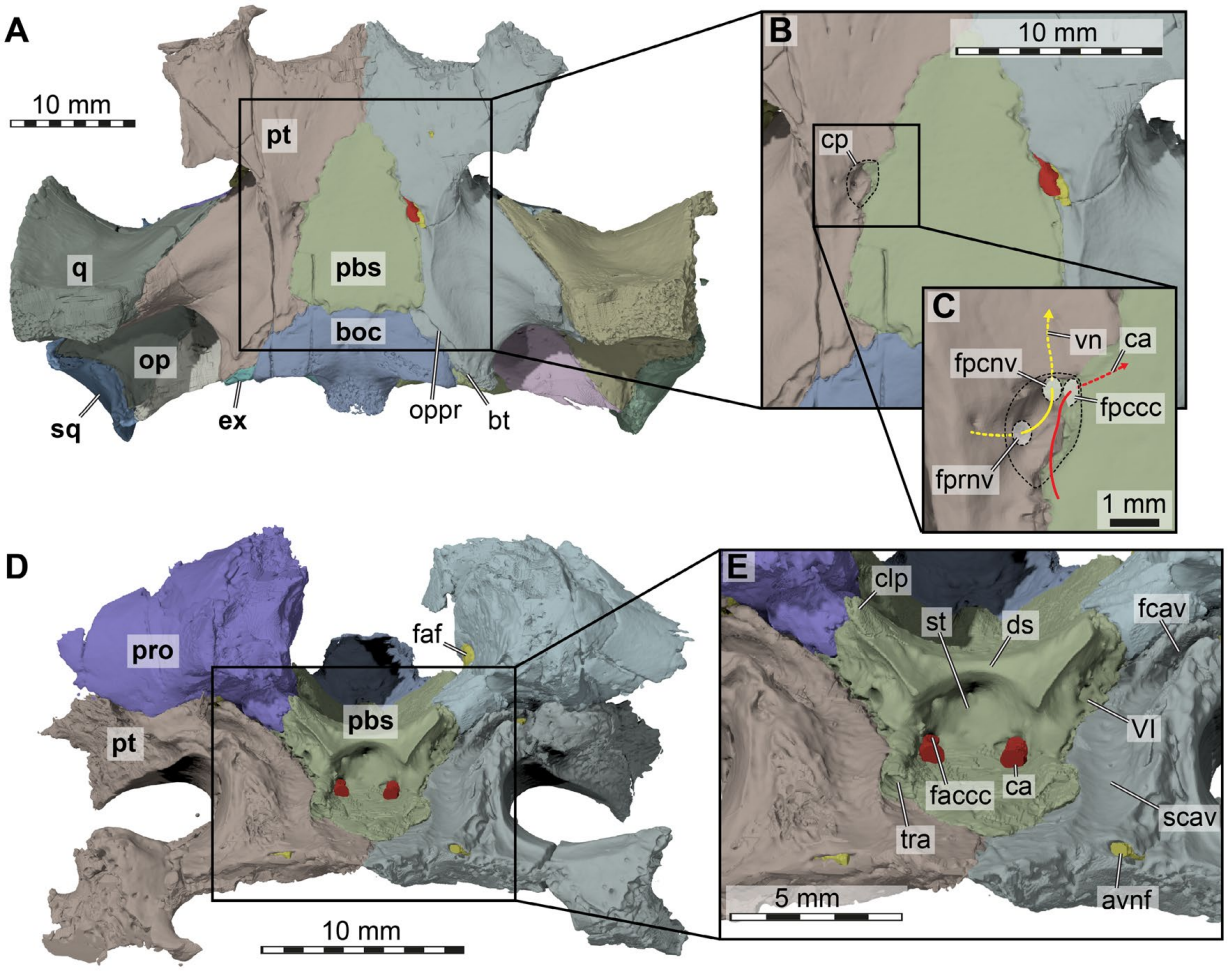
3D renderings of the basicranium of the holotype of *Saxochelys gilberti* (DMNH EPV.96000). **A** ventral view into basicranium. **B** close-up of parabasisphenoid region. **C** further close-up of right carotid pit. **D** anterodorsal view of partial basicranium. **E** close-up of anterior parabasisphenoid surfaces. Note that bones are labelled in bold font, and traits are labelled in regular font. avnf, anterior vidian nerve foramen; boc, basioccipital; bt, basal tuber; ca, cerebral artery; clp, clinoid process; cp, carotid pit; ds, dorsum sellae; ex, exoccipital; faccc, foramen anterius canalis carotici cerebralis; faf, fossa acustico-facialis; fcav, foramen cavernosum; fpccc, foramen posterius canalis carotici cerebralis; fpcnv, foramen posterius canalis nervi vidiani; fprnv, foramen pro ramo nervi vidiani; op, opisthotic; oppr, oblique posterior pterygoid ridge; pbs, parabasisphenoid; pro, prootic; pt, pterygoid; scav, sulcus cavernosus; sq, squamosal; st, sella turcica; tra, trabecula; VI, (anterior) foramen for the abducens (CN VI) nerve; vn, vidian nerve

*Pterygoid*. The pterygoids are completely preserved in DMNH EPV.96000 (Figs. 2B, 4, 5). The pterygoid is one of the largest bones of the skull and has the typical structures found in most turtles, namely a posterior process that forms the floor of the cavum acustico-jugulare, an anterior part that includes the transverse process and which contacts the anterior palatal region, and a dorsal crista pterygoidei which contributes to the lateral braincase wall.

The anterior part the pterygoid contacts the vomer anteromedially by overlapping its posterior aspect (Fig. 2B). Anteriorly both pterygoids contact one another along a short straight suture anterior to the parabasisphenoid and posterior to the vomer. The pterygoid further contacts the palatine anteriorly, along a posteriorly convex suture (Fig. 2B). Anterolaterally, the pterygoid forms a prominent transverse process (Figs. 2B, D, 4A). The anteromedial margin of this process forms parts of the margin of the foramen palatinum posterius (Fig. 2B). Lateral to the foramen, the transverse process of the pterygoid is broadly sutured with the maxilla, and is dorsally expanded to form a large anterolateral facet toward the jugal (Fig. 2B). Posterior to the jugal articulation, the transverse process forms a posteriorly triangular extension that projects into the subtemporal fossa (Fig. 4A). The lateral surface of the transverse process is expanded to a vertical plate, although the plate height narrows posteriorly toward the spiky end of the process (Figs. 2D, 4A). Dorsal to the jugal contact, the pterygoid also contacts the postorbital. This contact is unusual for turtles, but is broadly found within pleurodires (Gaffney, 1979a) and many paracryptodires (e.g., *Eubaena cephalica*: Rollot et al., 2018; *Arundelemys dardeni*: Evers et al., 2021; *Pleurosternon bullockii*: Evers et al., 2020;*Trinitichelys hiatti*: Rollot et al., 2022a; *Uluops uluops*: Rollot et al., 2021a).

Centrally, between the transverse process and the posterior process, the pterygoid of DMNH EPV.96000 is mediolaterally constricted to its narrowest extent (Fig. 2B). In this area, halfway along the suture with the parabasisphenoid, three openings into the skull are aligned in a recessed area, the ‘carotid pit’ (see Rollot et al., 2021a; Figs. 2B, 5A, B, C). The largest opening is for the cerebral carotid artery and is positioned on the anteromedial corner of the carotid pit, at the suture with the parabasisphenoid (Fig. 5C). Posterolateral to this foramen, there is a smaller foramen that can be identified as the canalis pro ramo nervi vidiani (Fig. 5C), which carries the vidian nerve. As this posterior vidian canal and the entry foramen for the cerebral artery are exposed, the condition of DMNH EPV.96000 is not like in other baenodds such as *Eubaena cephalica* (Rollot et al., 2018), but more similar to those of early baenids (e.g., *Arundelemys dardeni*: Evers et al., 2021; *Trinitichelys hiatti*: Rollot et al., 2022a). The vidian nerve of DMNH EPV.96000 enters the skull directly again via a small foramen in the anterolateral corner of the carotid pit, the foramen posterius canalis nervus vidiani (Fig. 5C). This foramen leads into a thin canal that extends through most of the pterygoid, and exits this bone at the anterior end of the base of the crista cranii (Fig. 5D, E). The ventral surface of the central region of the pterygoid is further lined by a shallow but sharp-edged ridge, which extends from the posterior process of the pterygoid laterally by the carotid pit to merge with the lateral process (Figs. 2B, 5A).

The posterior process of the pterygoid is extensive, and ventrally covers the entire cavum acustico-jugulare (Figs. 2B, 4C, D, 5A). It contacts the basioccipital posteromedially, the exoccipital posteriorly, and the medial surface of the articular process of the quadrate laterally. The contact between the pterygoid and the basioccipital extends along the entire length of the basioccipital. Between the quadrate and exoccipital contacts, the pterygoid forms the ventral margin of the fenestra postotica (Fig. 4C, D), which is confluent with the foramen jugulare posterius. The pterygoid fossa on the ventral surface of the posterior pterygoid process is shallow (Figs. 2B, 5A). Roughly parallel with the suture to the basioccipital, and just shortly removed from it, there is an obliquely trending, unusual ridge on the ventral pterygoid surface, which produces a step-like transition toward the basioccipital surface (Fig. 5A). The function of the ridge is unclear, but, as it is spatially close to the position of the basioccipital tuber, it is possible that the ridge is associated with musculature insertions.

Dorsally, the pterygoid contacts the prootic and forms the canalis cavernosus (Figs. 4D, 5D, E). The canalis cavernosus is thereby framed laterally, ventrally, and medially by the pterygoid. The lateral and medial walls of the canalis cavernosus are formed by dorsal ridges of the pterygoid, which are better preserved on the right side. The lateral ridge lies against the quadrate posteriorly, and is anteriorly formed as a low crista pterygoidei, which contacts the parietal (Fig. 4A). Between these contacts, the lateral ridge is notched to form the ventral margin of the trigeminal foramen. Anterolateral to the foramen, there is also the epipterygoid facet of the pterygoid, which is developed as a shallow groove on the lateral side of the bone (Fig. 4B). The second, more medially placed ridge of the dorsal surface of the pterygoid persists only for the length of the canalis cavernosus. The posterior part of the ridge forms a small sulcus for the hyomandibular nerve within the wall of the canalis cavernosus. In addition, the dorsal surface of the pterygoid also bears an opening into the vertically descending canalis pro ramo nervi vidiani.

*Supraoccipital*. Despite some fractures on the right side of the bone, the supraoccipital is well-preserved. It is a single, median bone that is exposed on the back of the skull (Figs. 2A, F, 3, 4C). The bone has a tiny contribution to the dorsal skull posterodorsally (Fig. 2A). The crista supraoccipitalis is extremely short, not reaching beyond the occipital condyle, and its posterior margin seems to be preserved as in life (Figs. 2F, 3, 4C). The supraoccipital roofs the foramen magnum ventrally and the cavum labyrinthicum ventrolaterally. On the left side, a small foramen aquaducti vestibuli is preserved (Fig. 3A), which forms an opening between the cavum labyrinthicum and cavum cranii. The supraoccipital contacts the parietal anteriorly and dorsally. Furthermore, it contacts the prootic anterolaterally, and the quadrate laterally (see quadrate for further comments), and the opisthotic and exoccipital posterolaterally.

*Exoccipital*. The exoccipitals are completely preserved in DMNH EPV.96000 (Figs. 2F, 3, 4C). The exoccipital forms the lateral wall of the cavum cranii and the lateral margins of the foramen magnum, and contacts the supraoccipital dorsally, opisthotic laterally, pterygoid ventrolaterally, and basioccipital ventromedially (Fig. 2F). A contact with the basisphenoid within the braincase is clearly absent (Fig. 3). An exoccipital-exoccipital contact is absent both in the dorsal and ventral part of the foramen magnum (Fig. 2F). The posteromedial process of the exoccipital, which in many turtles contributes to the occipital condyle, is extremely short in DMNH EPV.96000, so that the condyle is exclusively formed by the basioccipital (Figs. 2F, 4C). This contradicts statements of the original *Saxochelys gilberti* description by Lyson et al. (2019). Although this occipital condyle configuration is unusual for turtles generally (e.g., Gaffney, 1979a), and reportedly also for baenids (e.g., Gaffney, 1982; Lyson & Joyce, 2009a; Lyson et al., 2016, 2019), it does appear in a large number of recently described or re-described species. Among baenids, this occipital condyle configuration has been documented in *Trinitichelys hiatti* (Rollot et al., 2022a) and *Denazinemys nodosa* (Spicher et al., 2023), among non-baenid paracryptodires in *Uluops uluops* (Rollot et al., 2021a) and *Pleurosternon moncayensis* (Pérez-García et al., 2021), as well as in the potentially closely related *Kallokibotion bajazidi* (Martín-Jimenez et al., 2021). The occipital exposure of the exoccipital of DMNH EPV.96000 is relatively broad, and features two prominent foramina for the hypoglossal nervi rami (Figs. 2F, 4C). The lateral margin of the exoccipital is deeply notched around the “foramen” jugulare posterius, which is laterally confluent with the fenestra postotica (Figs. 2F, 4C). Below the fenestra, the exoccipital has a ventrolateral process that contacts the pterygoid and basioccipital in the region of the basioccipital tubercle (Figs. 4F, 5A). On its medial surface, the exoccipital contributes to the foramen jugulare anterius (Figs. 3, 4D), which connects the cavum cranii medially with the recessus scalae tympani, for which the exoccipital forms the posterior wall.

*Basioccipital*. The basioccipital is a single, median bone that is entirely preserved in DMNH EPV.96000 and is located at the posterior base of the skull (Figs. 2B, F, 3, 4C, 5A). The general shape of the basioccipital is triradiate in ventral view. The bone forms the occipital condyle and floors the posterior surface of the cavum cranii (Figs. 2F, 4C). It also forms the ventral aspect of the foramen magnum. The basioccipital, along with the exoccipital and the pterygoid, form the basioccipital tubercles (Fig. 5A). These project as pointed processes posterolaterally, and a ridge continues from the pointed tip of the process across the basioccipital towards the skull midline. The right and left ridge do not meet medially, and define a clear and sharply defined change of surface orientation between the posterior and ventral surfaces of the basioccipital. The basioccipital contacts the parabasisphenoid anteriorly along a transverse, straight suture. However, the external visible suture is caused by an underlapping process of the parabasisphenoid, so that the ventral suture between both bones assumes a more posterior position than the dorsal suture. The basioccipital also contacts the exoccipitals laterally and dorsally on a large contact surface. A basis tuberculi basalis is only very shallowly formed toward the contact area with the parabasisphenoid (Fig. 3).

*Prootic*. Both prootics are preserved in articulation (Figs. 3, 4, 5D), but the elements are damaged on both sides, particularly near their articulation with the parabasisphenoid. The damage on both sides is nearly symmetrical. The prootic is located in the posterior half of the skull and is exposed in the temporal fossa, in which it floors the anterior region and forms parts of the otic capsule and the foramen stapedio-temporale. It contacts the parietal anterodorsally, the supraoccipital posteromedially, the opisthotic posteriorly, the quadrate laterally, the pterygoid ventrally, and the parabasisphenoid anteroventrally. The externally exposed surface of the prootic forms a major part of the processus trochlearis oticum (Fig. 4A), as the prootic extends superficially over the quadrate along the anterior end of the upper temporal fossa. However, the trochlear process is extremely weakly developed, without forming a clear or even protruding concave surface of the coronar aponeurosis tendon of the adductor musculature.

The prootic forms the posterodorsal wall of the trigeminal foramen (Fig. 4A, B). The prootic contacts the parietal posterodorsal to the trigeminal foramen, and has a broad suture on its anteromedial margin with this bone. The prootic has a short anteroventromedial process, which contacts the parabasisphenoid posterior to the trigeminal foramen and medial to the foramen cavernosus (Fig. 5D, E). The latter foramen is formed by the prootic dorsally, and the pterygoid ventrally. The canal for the facial nerve opens on the medial surface of the prootic from within the fossa acustico-facialis, and traverses laterally through the bone (Fig. 5D). However, the canal does not open directly into the canalis cavernosus. Instead, the canal gets broader nearing the position of the canalis cavernosus, forming a fossa-like cavity within the prootic in which the geniculate ganglion must have been positioned (e.g., Rollot et al., 2021b). Here, the canal then splits into two separate canals. One is ventrally-directed, leaves the prootic and enters the pterygoid (without having entered the canalis cavernosus). This canal is the canalis pro ramo nervi vidiani for the vidian nerve, which exits in the carotid pit (see pterygoid; Fig. 5C). The second canal is posteriorly directed from the geniculate ganglion position, carries the hyomandibular ramus of the facial nerve, and then enters the canalis cavernosus (Rollot et al., 2021b). Near the medial dorsal pterygoid ridge, the prootic forms a posteriorly extending sulcus for the hyomandibular nerve, which persists until the canalis cavernosus merges with the cavum acustico-jugulare (Fig. 4D).

In the interior, the prootic houses large parts of the cavum labyrinthicum, and two foramina for the acoustic nerve open from the fossa acustico-facialis into the inner ear (Fig. 3A, B). One of these foramina is larger, and probably represents the merging of two separate foramina, as the acoustic nerve is expected to have three separate rami (e.g., Evers et al., 2019; Ferreira et al., 2022). Together with the opisthotic, the prootic of DMNH EPV.96000 forms the fenestra ovalis, but this opening remains ventrally open as there is no contact between the prootic and the processus interfenestralis of the opisthotic (Fig. 5D). Just lateral to the fenestra ovalis, the prootic bears a deep fossa, the posterior prootic recess (Evers & Joyce, 2020), on its posterior surface that faces the cavum acustico-jugulare (Fig. 5D). Lateral to the position of the posterior prootic recess, and dorsally with respect to the posterior foramen into the canalis cavernosus, the canalis stapedio-temporale opens between the prootic and quadrate (Fig. 5D). The canal extends vertically and exits dorsally in the foramen stapedio-temporale, which again is formed by the prootic and quadrate.

*Opisthotic*. Both opisthotics are well-preserved in DMNH EPV.96000 (Figs. 2F, 4C, D). The bone is positioned in the posterior part of the skull, forms parts of the temporal fossa and otic capsule, as well as the posterior margin of the fenestra postotica. The opisthotic contacts the exoccipitals ventromedially, supraoccipital dorsomedially, quadrate anterolaterally and squamosal laterally. It also broadly contacts the prootic anteriorly, but this contact is not visible on the skull surface. Contacts with the basioccipital and parabasisphenoid are absent.

The medial part of the opisthotic houses the posterior part of the cavum labyrinthicum. Ventrally, the opisthotic bears a thin processus interfenestralis (Fig. 5D), which is ventrally restricted and which does not contact the floor of the inner ear cavity. The processus interfenestralis bears a posterior opening, the fenestra perilymphatica, which opens anteroposteriorly from the cavum labyrinthicum into the recessus scalae tympani (Fig. 5D). The processus interfenestralis forms the anterior wall of the recessus scalae tympani, and also the posterior margin of the fenestra ovalis. At the base of the processus interfenestralis, the foramen for the glossopharyngeal nerve is visible on both sides (Fig. 5D). The hypoglossal canal enters the floor of the cavum labyrinthicum, so that there is another glossopharyngeal foramen medially within the opisthotic (see Gaffney, 1979a).

Posterolaterally, the opisthotic has a prominent paroccipital process, which lies posteriorly against the quadrate and extends laterally to contact the squamosal (Fig. 2F). The posterior margin of the paroccipital process is flattened to a ridge-like rim which projects slightly ventrally and forms the dorsal margin of the fenestra postotica. The posterior surface of the opisthotic above this margin is slightly concave, and serves as a muscle attachment area for neck muscles (Werneburg, 2011).

*Parabasisphenoid*. The parabasisphenoid is a single, median bone in the posterior half of the skull (Figs. 2B, 3, 5) and is completely preserved in DMNH EPV.96000. The bone has a general triangular shape when viewed in articulation within the basicranium in ventral view, whereby it is broadest posteriorly along the suture with the basioccipital, and narrows anteriorly between the pterygoids. The parabasisphenoid of DMNH EPV.96000 contributes to the carotid pit (Fig. 5A, B, C), which is midway along its contact with the pterygoid. From there, the canal for the cerebral arteries pierces the bone anterodorsomedially (Fig. 5C). Dorsolaterally, the parabasisphenoid contacts the prootic, but contacts with the exoccipital and opisthotic are absent internally.

The posterior part of the ventrally exposed triangular shape of the parabasisphenoid is formed by a thin, laminar extension of the bone that overlaps the anterior part of the basioccipital. Anteriorly, the parabasisphenoid gradually overlaps the pterygoids following the midline contact between both pterygoids with a flat rostrum basisphenoidale (Fig. 5D, E). The rostrum basisphenoidale is flanked on either side by rod-like, anteroposteriorly trending trabeculae, which contact the pterygoid in the medial margin of the sulcus cavernosus (Fig. 5D, E). The dorsum sellae is raised relatively highly with regard to the plate of the rostrum basisphenoidale, exposing a deep and relatively spacious sella turcica, in which the foramina anterior canalis carotici basisphenoidalis are widely separated at the lateral ends of the fossa (Fig. 5D, E). The posterior wall of the sella turcica is without a vertical ridge and is smooth. The dorsum sellae, although being relatively highly raised, does not form a dorsally projecting transverse ridge, but projects anteriorly slightly over the sella turcica (so that the dorsum sellae should be scored as being ‘low’ in the taxonomy of the commonly used phylogenetic character that describes the relative height of the sella; Fig. 5E). The clinoid processes are well-preserved as robust, but flattened, short processes that project principally dorsally. Anteromedially, the clinoid processes are associated with a ridge-like lamina that extends towards the margin of the sella turcica (Figs. 3A, 5E). Although these ridges form a well-developed anterolaterally exposed surface on the basisphenoid above the floor of the sulcus cavernosus, retractor bulbi pits are not formed (Fig. 5E). The anterior foramina for the abducens nerve lie in this anterolateral surface, and they are completely surrounded by bone of the parabasisphenoid (Fig. 5E). Posteriorly, the clinoid processes define a dorsal notch between the parabasisphenoid and prootic, which allows the trigeminal nerve stem to exit the cavum cranii and enter the cavum epiptericum (Fig. 3A; e.g., Evers et al., 2019). The dorsal surface of the parabasisphenoid bears a low but distinct basis tuberculi basalis, a median ridge in the posterior part of the bone that continues for a short distance onto the basioccipital (Fig. 3).

**Fig. 6:**
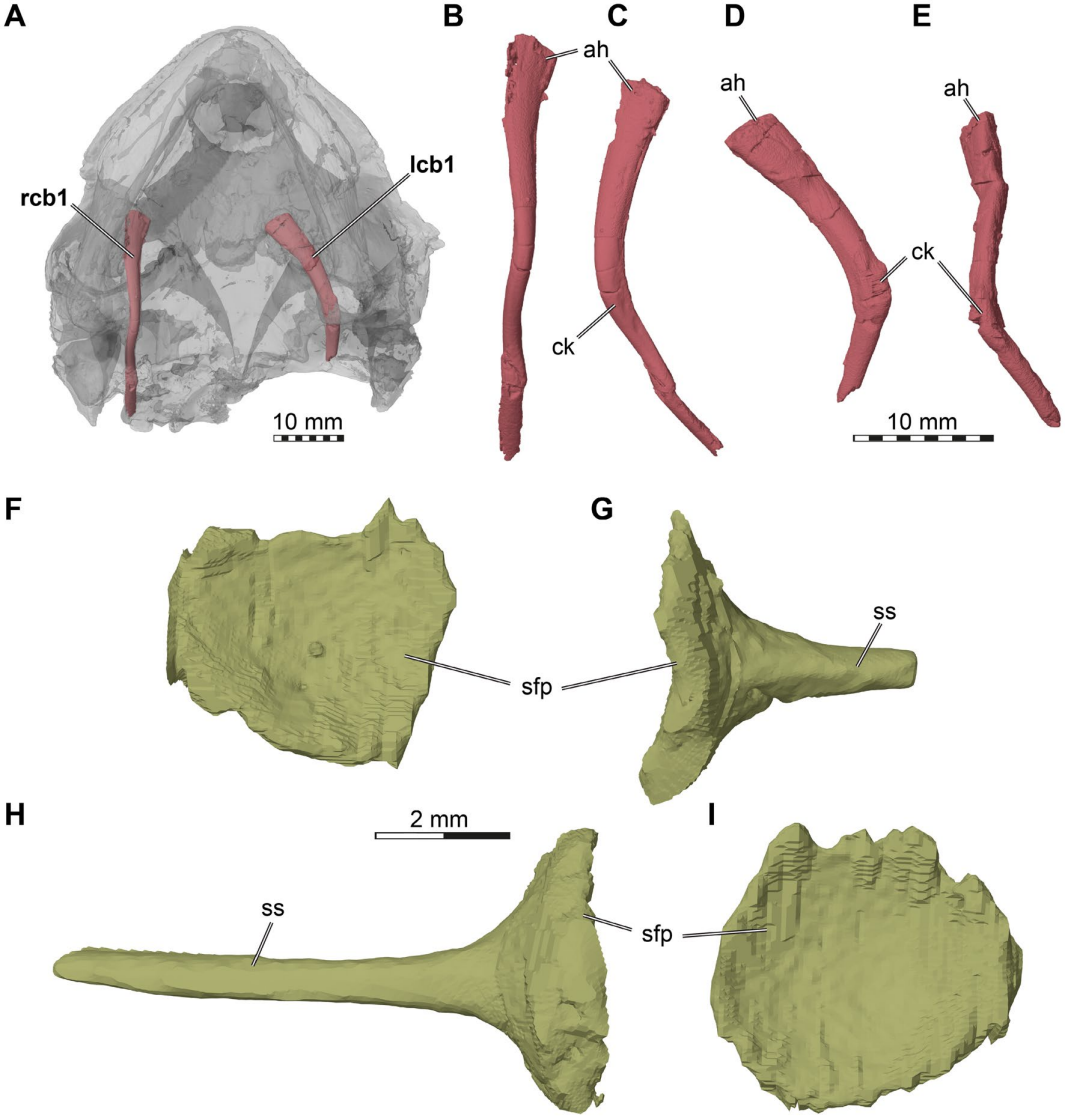
3D renderings of the hyoids and stapes of the holotype of *Saxochelys gilberti* (DMNH EPV.96000). **A** translucent cranium in ventral view showing position of preserved ceratobranchials. **B** right ceratobranchial I in ventral view.** C** right ceratobranchial I in medial view. **D** left ceratobranchial I in ventral view.** E** left ceratobranchial I in medial view. **F** left stapes in medial view (dorsal is up). **G** left stapes in anterior view. **H** right stapes in anterior view. **I** right stapes in medial view (dorsal is up). ah, articular head; ck, central kink; lcb1, left ceratobranchial I; rcb1, right ceratobranchial 1; sfp, stapedial footplate; ss, stapedial shaft

*Hyoids*. Two partial ceratobranchial I bones are preserved in DMNH EPV.96000 (Fig. 6A). These are commonly the only fossilized hyoid bones, even in well-preserved and articulated turtle crania (e.g., Joyce et al., 2021b), indicating that they may have been the only ossified hyoid elements of many turtles, including *Saxochelys gilberti*. The right ceratobranchial I of DMNH EPV.96000 is very long, extending from the level of the transverse process of the pterygoid anteriorly to the posterior tip of the squamosal posteriorly (Fig. 6A, B, C), suggesting the element is nearly complete. Anteriorly, both ceratobranchials I are mediolaterally slightly flattened and dorsoventrally slightly expanded, resulting in a broad anterior head (Fig. 6B, C, D, E). The anterior surface of this head is rounded, suggesting it represents the original articulation head for the hyoid body. Posteriorly, the ceratobranchial I narrows but retains an oval cross-section. At the level of the pterygoid fossa, the ceratobranchial becomes posterodorsally directed, resulting in a kink in its profile (Fig. 6C, D, E). The posterodorsally ascending part is yet again somewhat thinner, and develops a more circular cross-section. This rod-like appearance is maintained until its posterior end, where a recurved piece that is observed among extant turtles (e.g., Lemell et al., 2002) is missing, probably due to breakage.

*Stapes*. Both stapes are incompletely preserved in DMNH EPV.96000 (Fig. 6F, G, H, I), with the stapedial footplate intact on both sides. The stapes is preserved in a slightly disarticulated manner, whereby it was pushed into the inner ear cavity. The shape of the stapes is similar to that found more broadly in turtles, with a stapedial footplate as a rounded medial expansion with a concave medial surface and a stapedial rod that projects laterally toward the tympanum (Fig. 6F, G, H, I; e.g., Foth et al., 2019). The stapes is slightly more robust than recently reported for *Plesiobaena antiqua* (Smith et al., 2023), although this is difficult to ascertain due to the incomplete preservation of the stapedial rod in DMNH EPV.96000.

**Fig. 7:**
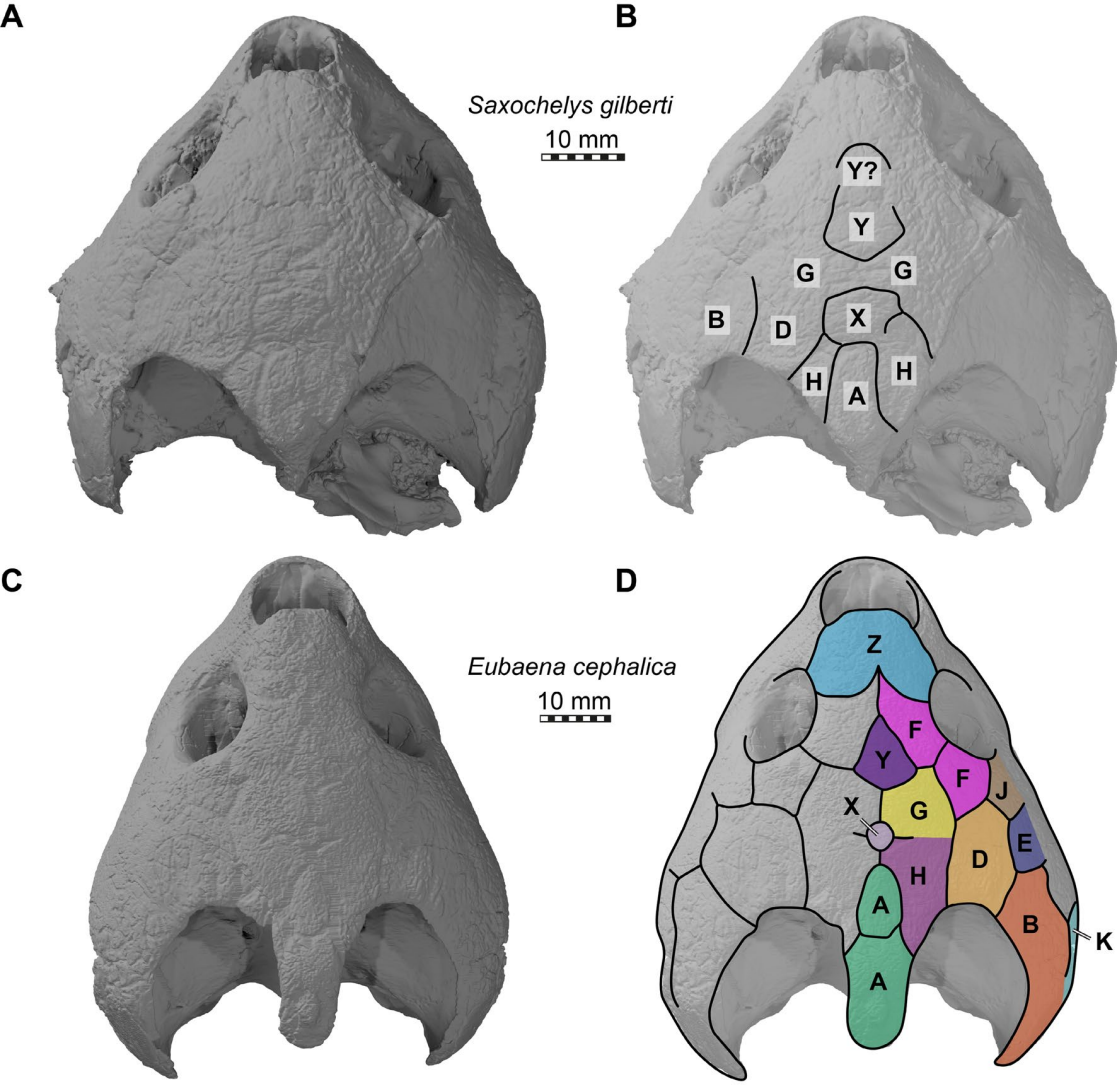
3D renderings of the crania of the holotype of *Saxochelys gilberti* (DMNH EPV.96000) and *Eubaena cephalica* (DMNH EPV.96004). **A** cranium of DMNH EPV.96000 in dorsal view and low light setting. **B** as **A** with interpretative line drawing of scute sulci. **C** cranium of DMNH EPV.96004 in dorsal view and low light setting. **D** as **C** with interpretative line drawing of scute sulci. Note that capital letters on skulls represent scute identity

*Cranial scutes.* The scute sulci of DMNH EPV.96000 are hard to discern, and only a few scutes can be traced on the skull roof (Fig. 7A, B). Scute identification is also difficult, as few rigorous homology criteria for scutes exist (e.g., Evers et al., 2021). Scutes are thus largely identified based on comparative observations with taxa for which the scute patterns is known. The only baenid for which a nearly complete scute pattern has been published is *Arundelemys dardeni* (Evers et al., 2021). The identification and scute homology for that study was developed based on comparisons with other stem turtles, and is largely based on prior work by Sterli and de la Fuente, (2013). Rollot et al. (2018) published a partially inferred scute pattern for *Eubaena cephalica*. We here re-interpreted the scute sulci interpretation of Rollot et al. (2018) based on investigations of 3D models generated from CT data. In low light settings and moving the cranium around, it is possible to identify scute sulci that cover the entire specimen, so that we can here produce a complete scute interpretation for the taxon. Our scute identifications for *Saxochelys gilberti* are based on comparisons with both *Arundelemys dardeni* and *Eubaena cephalica*. As the latter taxon has a more complete scute pattern than DMNH EPV.96000, we first briefly describe the pattern found in *Eubaena cephalica*.

*Eubaena cephalica* (DMNH 96004) has a supernumerary median midline scute at the posterior end of the cranium (Fig. 7D). We herein define this to be a second A scute. The posterior A scute covers the supraoccipital and posterior interparietal area. The other A scute is somewhat smaller, and extends over the interparietal area (Fig. 7D). Anterior to this, but slightly removed from the anterior A scute by a midline contact of the H scutes, *Eubaena cephalica* has a small, round X scute (Fig. 7D). Anterior to the X scute, there is a midline contact between paired G scutes (Fig. 7D). At the level of the posterior part of the orbits, there is a roughly triangular Y scute in the midline, which tapers anteriorly toward a midline contact of paired anterior F scutes (Fig. 7D). The anterior region of the skull roof is covered by a large Z scute, which has the shape of an inverted heart (Fig. 7D). The orbit is lined by two F scutes and a J scute in the jugal area (Fig. 7D). The G scute covers the area of the skull between the F scute and the X scute, and is followed posteriorly by the H scute, which extends into the margin of the upper temporal emargination (Fig. 7D). The D scute lies laterally adjacent to both the H and G scutes, and contacts the second F scute anteriorly (Fig. 7D). The J scute is posteriorly followed by an E scute, which itself is posteriorly followed by a large B scute that covers the squamosal area. Ventral to the B scute, there is a K scute (Fig. 7D).

*Saxochelys gilberti* (DMNH EPV.96000) has two midline scutes that are relatively clearly evident on the posterior end of the skull (Fig. 7A, B). The posteriormost overlaps the supraoccipital and posterior ends of the parietals. It is anteroposteriorly longer than mediolaterally broad, and its lateral margins are anteromedially directed away from the upper temporal emargination. This scute is certainly an A scute (Fig. 7B; Evers et al., 2021). The A scute is anteriorly met by a second midline scute, which is mediolaterally broader than anteroposteriorly long (Fig. 7A, B). It is restricted to the interparietal area and contacts an elongate scute posterolaterally, which lies laterally against the A scute. This overall pattern is consistent with an interpretation of the second midline scute being the X scute (Fig. 7B): The X scute is also medially broader than the A scute in *Arundelemys dardeni* (Evers et al., 2021), and contacts the H scute that is laterally adjacent to the A scute in both *Arundelemys dardeni* (Evers et al., 2021) and *Eubaena cephalica* (Fig. 7D). It is unlikely that the second midline scute of DMNH EPV.96000 represents a supernumerary A scute (as is found in *Eubaena cephalica*), namely due its broad shape compared to the posterior, definitive A scute. Another midline scute can be seen slightly anterior to the X scute (Fig. 7A, B). However, this scute is removed from the X scute, suggesting they were separated by a set of paired scutes (G scutes; Fig. 7B). This third midline scute likely represents the Y scute (Fig. 7B). Evidence for this interpretation comes from its position in the center of the skull at the posterior level of the orbit, where both *Arundelemys dardeni* (Evers et al., 2021) and *Eubaena cephalica* (Fig. 7D) also have their Y scutes. In addition, the posteriorly convex shape of the Y scute seen in DMNH EPV.96000 is also found in *Eubaena *cephalica (Fig. 7B, D). Anterior to the Y scute of DMNH EPV.96000, there is another incomplete trace of a midline scute (Fig. 7A, B). As the anterior limits of the Y scute and the posterior limits of this scute are not clear, it is possible that both belong to the same (Y)scute, which is usually the most anterior of the unpaired median skull scutes that does not extend into the external naris (Evers et al., 2021). A paired scute can also be seed adjacent to the A scute of DMNH EPV.96000 (Fig. 7A, B). This scute is mediolaterally narrow, and anteriorly framed by the possible X scute. Due to its topological position, we identify this as an H scute (Fig. 7B). Its shape is irregular on the right side. The H scutes of DMNH EPV.96000 do not meet on the midline due to the broad shape of the X scute and a contact between A and X scutes. This is also the case in *Arundelemys dardeni* (Evers et al., 2021), but contrasts with the condition of *Eubaena cephalica* (Fig. 7D). On the left skull side, there is yet another incomplete scute traceable from the upper temporal margin anteriorly in DMNH EPV.96000 (Fig. 7A, B). This scute possibly delimits the D scute medially from the B scute laterally, and its position is roughly matched by that in both *Arundelemys dardeni* and *Eubaena cephalica*.

**Fig. 8:**
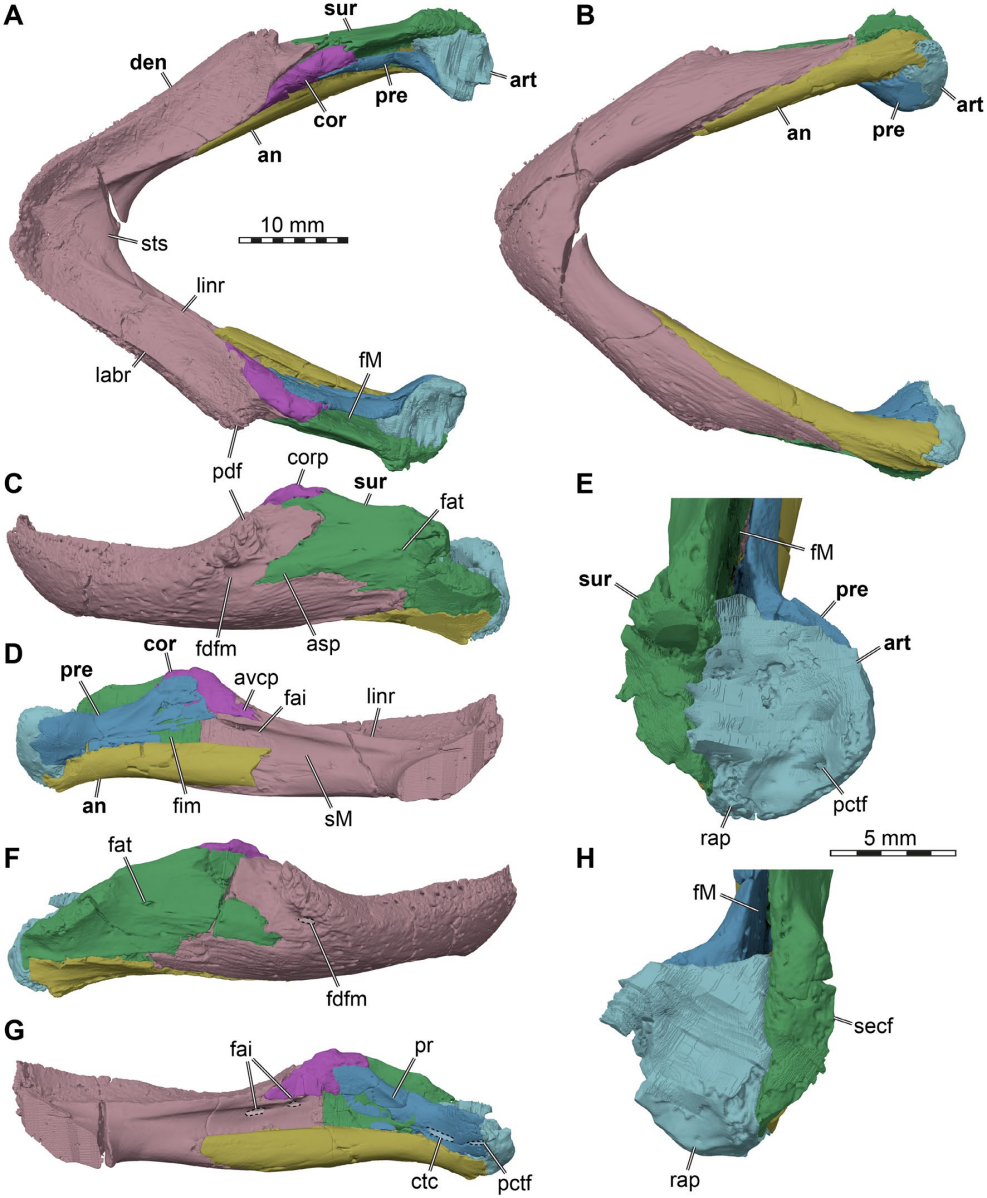
3D renderings of the mandible of the holotype of *Saxochelys gilberti* (DMNH EPV.96000). **A** dorsal view. **B** ventral view. **C** left mandibular ramus in lateral view. **D** left mandibular ramus in medial view. **E**, close-up of articular area of left mandibular ramus. **F** right mandibular ramus in lateral view. **G** right mandibular ramus in medial view. **H** close-up of articular area of right mandibular ramus. Note that bones are labelled in bold font, and traits are labelled in regular font. an, angular; art, articular; asp, anterior surangular process; avcp, anteroventral coronoid process; cor, coronoid; corp, coronoid process; ctc, chorda tympani canal (broken); den, dentary; fai, foramen alveolare inferius; fat, foramen auriculotemporalis; fdfm, foramen dentofacialis major; fim, foramen intermandibulare medius; fM, fossa Meckelii; labr, labial ridge; linr, lingual ridge; pctf, posterior chorda tympani foramen; pdf, processus dentofacialis; pr, prearticular ridge; pre, prearticular; rap, retroarticular process; secf, surangular ectocondylar flange; sM, sulcus Meckelii; sts, symphyseal tongue shelf; sur, surangular

*Dentary*. The dentaries of DMNH EPV.96000 are fused to a single element, which is well-preserved in the specimen (Fig. 8). It forms major parts of the mandible, including the triturating surfaces. The dentary contacts the angular medially and ventrally, the surangular posteriorly, and the coronoid posterodorsally. A contact with the prearticular is present on the left side only, in the form of a point contact below the position of the coronoid (Fig. 8D). The contact may be broken away on the right side. A contact with the articular, which was suspected to be present by Lyson et al. (2019), is absent (Fig. 8B).

The dentary is generally robust. As in many other baenids (Gaffney, 1982), the triturating surface of DMNH EPV.96000 is broadest posteriorly near the coronoid process, and narrows anteriorly toward the symphysis (Fig. 8A). The symphysis is posteroventrally expanded beyond the actual triturating surface part, again as in baenids more generally (Gaffney, 1982). This feature is seen in a variety of turtles (Evers et al., 2022), but the respective shelf has not been named. We herein refer to it as the symphyseal tongue shelf (Fig. 8A). The labial ridge is very low posteriorly, but becomes more defined anteriorly along the dentary ramus, where it forms a sharp-edged margin to the jaw (Fig. 8A, D, G). At the symphysis, the labial ridge forms a strongly upcurved tip of the beak, but the tip remains well-rounded and is not developed as a pointed hook. The lingual margin is medially expanded over the main part of the dentary ramus, forming a sharped edged margin (Fig. 8A, D, G). As this margin is not dorsally raised to a true lingual triturating ridge, this expansion forms more of a lingual platform as also seen in many extant turtles of various clades (Evers et al., 2022). The sulcus Meckelii at the medial surface of the dentary ramus is relatively shallow, and does not invade the symphysis anteriorly (Fig. 8A, D, G). The foramen alveolare inferius is exposed in medial view of the mandibular ramus, and divided into two openings on the right side (Fig. 8G), but not on the left (Fig. 8D). Posterolaterally, the dentary has a distinct processus dentofacialis (Fig. 8A, C; e.g., Evers et al., 2022; dentary tubercle of Lyson & Joyce, 2009a, 2009b; Lyson et al., 2019). This process can also be observed in several other baenodds, such as *Plesiobaena antiqua*, *Stygiochelys estesi* and *Palatobaena bairdi* (Gaffney, 1982), although it can also be strongly reduced as in *Baena arenosa* (Gaffney, 1982) or entirely absent as in *Chisternon undatum* (Gaffney, 1982). As this feature has so far not been used in baenid systematics, this variation may be used for such purposes in the future. The foramen dentofaciale majus is a mid-sized foramen in DMNH EPV.96000, which opens ventral to the processus dentofacialis, at the anterior end of the adductor fossa (Fig. 8C, F), which largely lies on the surangular. The dentary is posteriorly forked in DMNH EPV.96000 and receives an anterior surangular process (Lyson et al., 2019), which differs from the surangular-dentary articulation of other baenodds for which the sutures have been indicated, i.e., *Palatobaena bairdi* (Gaffney, 1982), *Palatobaena cohen* (Lyson & Joyce, 2009b), and *Peckemys brinkman* (Lyson & Joyce, 2009a).

*Surangular*. The surangulars are well-preserved in DMNH EPV.96000 (Fig. 8). The surangular forms the posterolateral portion of the mandible and contacts the dentary anteriorly, the coronoid anterodorsally and anteromedially, the angular ventrally and the articular posteromedially. Large parts of the lateral surface of the surangular are exposed in DMNH EPV.96000 (Fig. 8C, F). There is a prominent anterior process that inserts into a respective forked notch of the dentary that is unusual for baenids (see dentary) (Fig. 8C, F) and also absent in pleurosternids (Evers, 2023), but which appears in several groups of extant turtles, particularly chelonioids and some testudinids (Evers et al., 2022). The adductor fossa of DMNH EPV.96000 on the lateral surface of the surangular is shallow and inconspicuous. The surangular is laterally bulged along its posterior end to a prominent surangular ectocondylar flange (Evers et al., 2022) that forms parts of the articulation facet (Fig. 8E, H). Just anterior to the surangular ectocondylar flange is a small-sized foramen auriculotemporalis on the lateral surangular surface (Fig. 8C, F). The respective canal is anteromedially directed and connects to the fossa Meckelii, of which the surangular forms the lateral wall and the dorsal margin to its entry foramen. A dorsal surangular foramen (Evers et al., 2022) is absent. The dorsal margin of the surangular along the opening into the fossa Meckelii is raised dorsally above the level of the medial margin formed by the prearticular (Fig. 8D, G). Although also apparent in other baenids (Gaffney, 1982), a similar arrangement is otherwise primarily known from pleurosternids (Evers, 2023) and pleurodires (Evers et al., 2022).

*Coronoid*. Both coronoids are preserved in DMNH EPV.96000 (Fig. 8). The coronoid is a small and inconspicuous bone on the inner side of the central region of the mandible. It contacts the dentary anterolaterally, the surangular laterally and the prearticular posteromedially. The coronoid forms an extremely low, widely rounded coronoid process (Fig. 8C, D, F, G) that contrasts with the dorsally projecting slightly pointed coronoid process of most other baenids such as *Eubaena cephalica* (Gaffney, 1982), *Baena arenosa* (Gaffney, 1982), *Chisternon undatum* (Gaffney, 1982), *Neurankylus torrejonensis* (Lyson et al., 2016), *Arvinachelys goldeni* (Lively, 2015), or *Palatobaena bairdi* (Gaffney, 1982) and is more similar to pleurosternids (Evers, 2023). The coronoid of DMNH EPV.96000 is wedged between the anterodorsal process of the prearticular and the surangular, hereby separating these bones anteriorly and minorly contributing to the margin of the dorsal foramen into the fossa Meckelii (Fig. 8A). Posterior processes to either side of the dorsal foramen into the fossa Meckelii are absent. Anteroventrally, the coronoid has a process that slots into the dentary along its posteromedial part of the lingual jaw margin (Fig. 8D, G), but this process is only expressed on the medial jaw surface and does not contribute to the triturating surface dorsally. A coronoid foramen (sensu Evers & Benson, 2019, see also Evers et al., 2022) is absent.

*Prearticular*. Prearticulars are preserved on both sides of DMNH EPV.96000 but are more fragmented than the other bones of the mandible (Fig. 8A, B, D, E, G, H). The prearticular is a flat and elongated bone on the medial surface of the mandible. It contacts the coronoid anteromedially, articular posteromedially, and angular ventrally. The left prearticular also has a point contact with the dentary (Fig. 8D), which is not preserved on the right side (Fig. 8G). The prearticular of DMNH EPV.96000 only has an anterodorsal process (i.e., no anteroventral process along the angular; Evers et al., 2022), which diverges dorsally away from the angular to contact the coronoid (Fig. 8D, G). On the medial surface of the anterodorsal process, there is a weak but distinct ridge that roughly parallels the margin of the dorsal foramen into the fossa Meckelii (Fig. 8G). Together with the angular and dentary, the prearticular forms the medial intermandibular foramen, which is the large opening from the fossa Meckelii into the medially uncovered sulcus Meckelii (Fig. 8D, G). Along its contact with the angular, the prearticular of many turtles forms the posterior intermandibular foramen. In DMNH EPV.96000, there is no positive evidence for the former presence of this foramen, but the area is slightly crushed, so the absence of the foramen cannot be known with certainty from this specimen, but it appears to be present in better preserved specimens (Lyson et al., 2019; see angular below for further comments). The prearticular also forms the medial wall of the fossa Meckelii, and the medial margin to the large dorsal opening into this space (Fig. 8A, E, H). A contact with the surangular is absent to both the anterior and posterior side of the dorsal foramen into the fossa Meckelii (Fig. 8A, E, H). The posterior end of the prearticular is medially strongly angled away from the plane of the anterior parts of the element (Fig. 8B, E, H). This is because the posterior part of the prearticular wraps around the mediolaterally broad articular. Hereby, the prearticular is excluded from contributing to the articulation surface for the quadrate (Fig. 8E, H). Ventrally to the articulation surface, the medial surface of the right prearticular shows a shallow canal that is partially broken, and appears to be for the chorda tympani nerve (Fig. 8G). This nerve either enters the mandible via the articular or the prearticular in turtles (Evers et al., 2022), and DMNH EPV.96000 seems to show both conditions, with the left foramen being positioned within the articular (see articular below; Fig. 8E).

*Angular*. Both angulars are preserved in DMNH EPV.96000 (Fig. 8), but the anterior end of the left element is broken off. It is an elongate bone of the mandible that is positioned along the medioventral surface of the mandibular ramus. It contacts the dentary, prearticular, articular and surangular. The angular of DMNH EPV.96000 is relatively prominent compared to that of many other turtles (Evers et al., 2022). It is relatively deep dorsoventrally along its anterior process (Fig. 8D, G), which in many turtles tapers to a thin spike anteriorly (Evers et al., 2022). In DMNH EPV.96000, the anterior process remains relatively constant in depth, and the anterior end is broadly rounded below the sulcus Meckelii (Fig. 8G). The posterior end of the angular is also large: it is broadly exposed on the lateral and medial surfaces of the posterior end of the jaw (Fig. 8C, D, F, G), and forms a broad ventral buttress to the overlying articular (Fig. 8B). Most of the dorsal margin of the angular is unbound by bone due to the absence of a splenial (see below), as the anterior process lies medially against the dentary and forms the ventral margin of the sulcus Meckelii. Only posteriorly is the dorsal margin of the angular contacted by the prearticular. If present, the posterior intermandibular foramen (foramen intermandibular caudalis of Gaffney, 1972b) lies in this suture (Evers et al., 2022). Although DMNH EPV.96000 bears no direct evidence for the presence of a posterior intermandibular foramen, Lyson et al. (2019) report its presence in *Saxochelys gilberti* based on DMNH EPV.98816, and this can be verified from their photographic evidence. The absence of the foramen in DMNH EPV.96000 could either be attributed to taphonomy, as the overlying prearticular is quite fragmented, or it demonstrates individual variation of this feature within *Saxochelys gilberti*.

*Articular.* The articulars are well-preserved bones that are positioned at the back of the mandible, forming the majority of the jaw articulation surface (Fig. 8). The articular is wedged between the prearticular anteromedially and the surangular laterally (Fig. 8E, H), and is ventrally buttressed by the angular (Fig. 8B). A contact with the dentary is absent (contra suggestions by Lyson et al., 2019). The articular is dorsally broad and nearly rounded, forming a posterodorsally exposed surface that faces the quadrate when the mandible is articulated with the cranium. The articular is centrally depressed, and raised along its medial and lateral margins (Fig. 8E, H). The medial margin forms a slightly protruding vertical ridge together with the surangular, which separates the articulation surface into medial and lateral subfacets. Medially, the margin of the articular is more strongly raised and slightly overturned, forming a small, lip-like retroarticular process (Fig. 8E, H). In this posteromedial region of the bone, there is a prominent posterior chorda tympani foramen (Evers et al., 2022), which is only clearly visible on the left side (Fig. 8E). On the right side, the canal for the chorda tympani nerve seems to instead lie within the prearticular (Fig. 8G).

*Splenial*. Splenials are plesiomorphically present in turtles (Gaffney, 1990), and known from pleurosternids as the sister group of baenids (Evers, 2023). They are also known for a range of baenids and have explicitly been documented for *Peckemys brinkman* (Lyson & Joyce, 2009a), *Palatobaena cohen* (Lyson & Joyce, 2009b), *Palatobaena bairdi* (Gaffney, 1982), *Eubaena cephalica* (Gaffney, 1982), and *Stygiochelys estesi* (Gaffney, 1982). However, they are also demonstrably absent in several taxa, particularly *Baena arenosa* and *Chisternon undatum* (Gaffney, 1982). Here, we confirm the observations of Lyson et al. (2019) that the splenial is absent in *Saxochelys gilberti* (Fig. 8). The specimen described here, DMNH EPV.96000, unambiguously lacks splenials, as the dorsal surface of the angular and ventral surface of the dentary along the sulcus Meckelii show smooth bone surfaces without any trace of a potential splenial facet (Fig. 8D, G). As such, the fossa Meckelii is relatively short in *Saxochelys gilberti*, and the sulcus Meckelii elongated. An additional consequence is the free exposure of the foramen alveolare inferius on the medial surface of the dentary (see above).

**Fig. 9:**
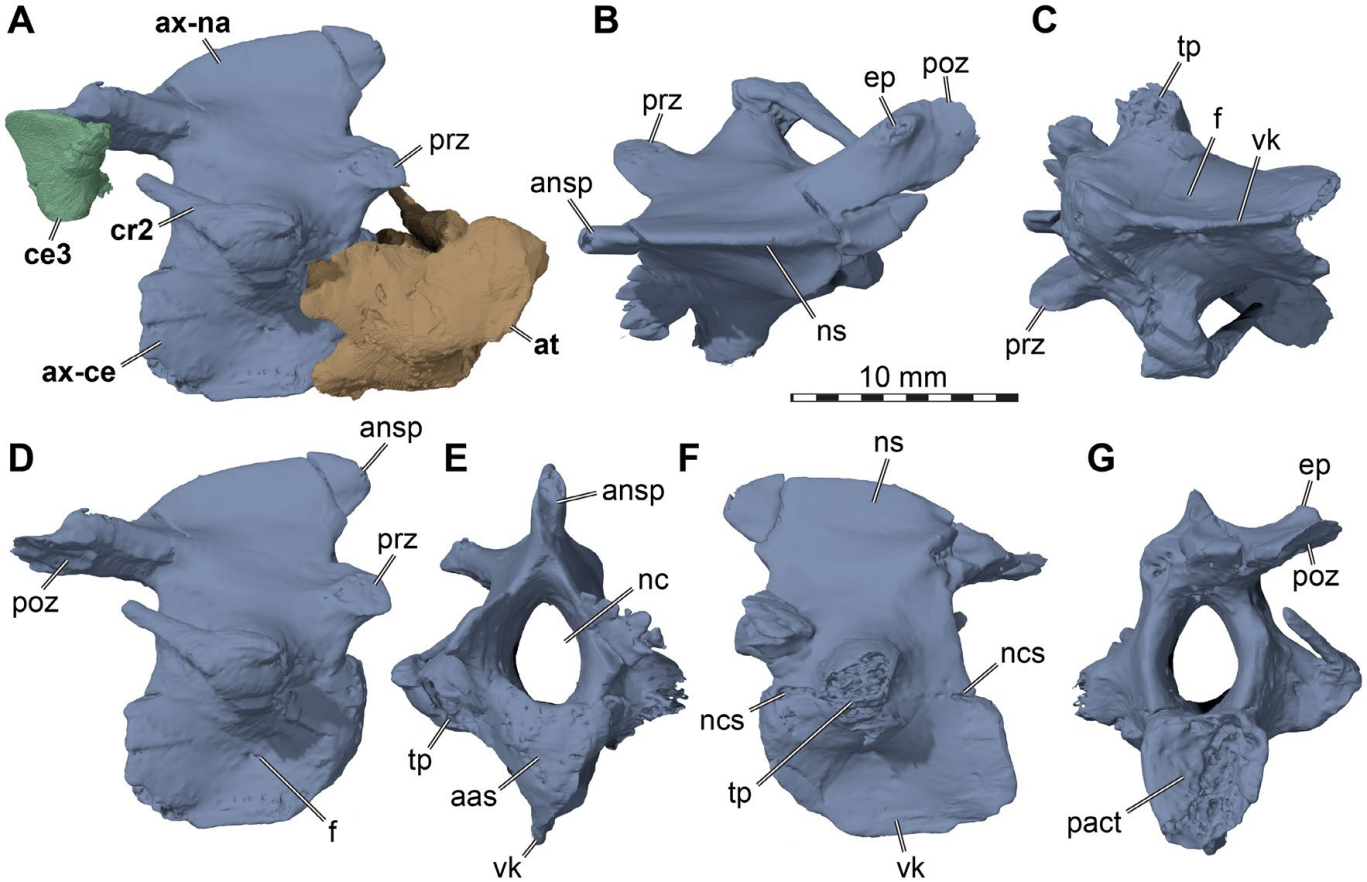
3D renderings of the cervical vertebrae of the holotype of *Saxochelys gilberti* (DMNH EPV.96000). **A** first three preserved cervical vertebrae in right lateral view. **B** axis in dorsal view (anterior to left). **C** axis in ventral view (anterior to left). **D** axis in right lateral view. **E** axis in anterior view. **F** axis in left lateral view. **G** axis in posterior view. Note that bones are labelled in bold font and traits in regular font. aas, anterior articulation surface; ansp, anterior neural spine process; at, atlas; ax-ce, centrum of axis; ax-na, neural arch of axis; ce3, cervical vertebra 3; cr2, cervical rib of axis; ep, epipophysis; f, foramen; nc, neural canal; ncs, neurocentral suture; ns, neural spine; pact, posterior articulation cotyle; poz, postzygapophysis; prz, prezygapophyses; tp, transverse process; vk, ventral keel

*Anterior cervical vertebrae.* The three most anterior elements of the vertebral column are preserved in DMNH EPV.96000: the atlas, the axis, and a small part of the third cervical vertebra (Fig. 9). The atlas and the third cervical vertebra are crushed and poorly preserved (Fig. 9A), so that we cannot comment on their morphology. In contrast, the axis is well-preserved (Fig. 9).

The axial centrum and neural arch are sutured, but the sutures can be traced in the CT scan (Fig. 9F). The intercentral articulations are platycoelous anteriorly (i.e., anteriorly flat; Fig. 9E), but clearly opisthocoelous posteriorly (i.e., concave posteriorly; Fig. 9G). The vertebral centrum is principally hour-glass shaped between these articulation surfaces (Fig. 9C), but this is ventrally somewhat obscured by the presence of a deep keel ventrally (Fig. 9C, D, E, F). The keel is convex along its ventral margin, and thus deepest in a central position of the vertebra (Fig. 9D, F). On each side, there is a small nutrient foramen at the base of the keel toward the hourglass-shaped main body of the centrum (Fig. 9C, D). The parapophyses and transverse processes seem to form a single unit, as no separate parapophyses are evident (Fig. 9F). The complex formed by these structures is commonly only referred to as the transverse process in turtles. This transverse process forms a large, laterally projecting, bump-like process that is quite short laterally (Fig. 9C, F). On the right side, a small cervical rib is still attached to the transverse process (Fig. 9A, B, D). The rib has a cup-shaped rib head, which articulates with its concave medial side to the rounded diapophysis of the transverse process. Posteriorly, the rib forms a rod-like process which is relatively short but likely broken distally (Fig. 9D).

The neural arch is dorsally taller than the centrum in DMNH EPV.96000 (Fig. 9D, E, F, G). Overall, the axis is dorsally taller than it is anteroposteriorly long. The neural canal is nearly twice as high as it is wide (Fig. 9E, G). The prezygapophyses are anteriorly projecting, yet short processes that emerge from the lateral arch that frames the anterior opening of the neural canal (Fig. 9A, D). The prezygapophyseal articulation facets are steeply vertically oriented so that they face strongly dorsolaterally, and not principally dorsally (Fig. 9D, E). The neural spine is formed as a vertical lamina that expands over the entire anteroposterior length of the neural arch (Fig. 9A, B, D, F). Anteriorly, this sheeted process overhangs the neural canal opening (Fig. 9B). The dorsal margin of the neural spine is convexly curved, and ends posteriorly between the postzygapophyses (Fig. 9D, F). These assume a position dorsally well above the level of the prezygapophyses, and are much longer than their anterior pendants. Only the right postzygapophysis is completely preserved in DMNH EPV.96000 (Fig. 9B, G). It bears a weak epipophysis dorsolaterally (Fig. 9B, G), and its articulation surface is strongly ventrally oriented and spoon-shaped.

A partial right prezygapophyses of the third cervical vertebra is still attached to the right axial postzygapophysis (Fig. 9A). The small piece allows us to say the prezygapophyseal processes of the third cervical are more prominent and longer than those of the axis, and the articular facets are strongly dorsally inclined, contrary to those described for the axis above.

### Phylogenetic results

**Fig. 10:**
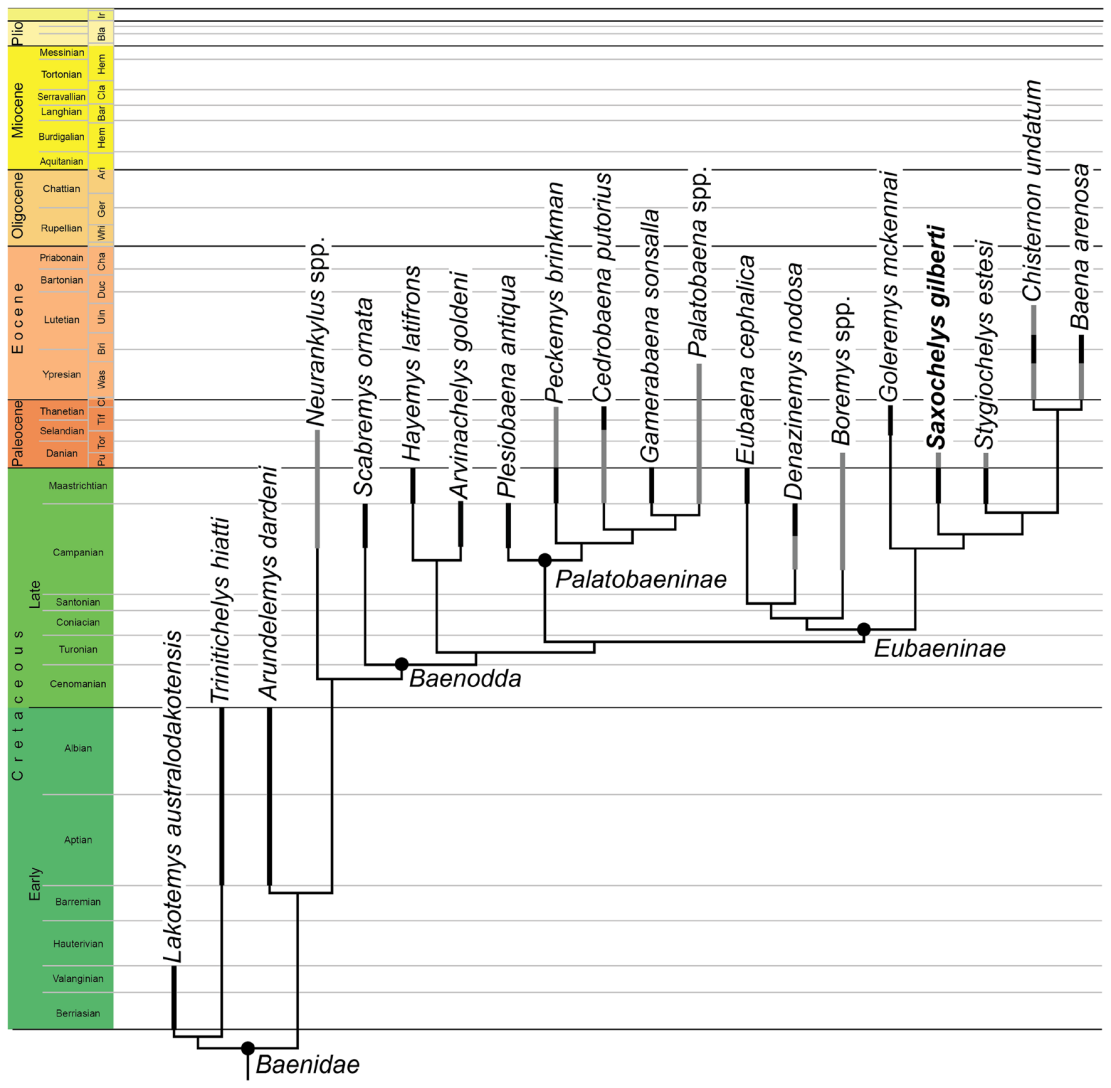
Strict consensus tree of 35 MPTs retained in the phylogenetic analysis, with stratigraphic ranges for taxa indicated by boxes along terminals. Note that black boxes indicate the age range of type material, whereas grey lines represent range extensions based on referred material. Age ranges are drawn over the complete duration of stages

The parsimony analysis resulted in 34 most parsimonious trees (MPTs) found with a step length of 371 character-state transitions after the initial search. An additional round of TBR branch swapping recovered one addition MPT, resulting in a final number of 35 MPTs. Despite the change of eight character-states with regard to the matrix of Spicher et al. (2023), the topological results are the same as those reported in that study (Fig. 10): *Saxochelys gilberti* is found as an eubaenine that is the sister taxon to a clade consisting of *Chisternon undatum*, *Baena arenosa*, and *Stygiochelys estesi*.

## Discussion

### New observations based on CT data

This study adds to the increasing number of paracryptodiran cranial re-descriptions based on CT data (e.g., *Arundelemys dardeni*: Evers et al., 2021; *Pleurosternon bullockii*: Evers et al., 2021; *Uluops uluops*: Rollot et al., 2021a; *Trinitichelys hiatti*: Rollot et al., 2022a; *Lakotemys australodakotensis*: Rollot et al., 2022b; *Denazinemys nodosa*: Spicher et al., 2023; *Plesiobaena antiqua*: Smith et al., 2023; *Glyptops ornatus*: Evers, 2023; *Saxochelys gilberti*: this study). Most of these studies contradict details found in the original descriptions that were based on traditional examination of specimens (e.g., *Arundelemys dardeni*: Lipka et al., 2006; *Pleurosternon bullockii:* Evans & Kemp, 1975; *Uluops uluops*: Carpenter & Bakker, 1990; *Trinitichelys hiatti*: Gaffney, 1972a; *Lakotemys australodakotensis*: Joyce et al., 2020a; *Denazinemys nodosa*: Lucas & Sullivan, 2006; Lively, 2016; *Plesiobaena antiqua*: Brinkman, 2003; Lyson & Joyce, 2009a; *Glyptops ornatus*: Gaffney, 1979b). This highlights the usefulness of CT data and bone-by-bone segmentations for the study of fossil turtle morphology and systematics. Here, we list the most important differences that we found in DMNH EPV.96000 with regard to the original *Saxochelys gilberti* description (Lyson et al., 2019).

Contrary to Lyson et al. (2019), the 3D models of DMNH EPV.96000 indicate that a nasal-maxilla contact was absent, and that both bones are instead separated by a small posterodorsal notch in the margin of the external naris. We also find, contrary to Lyson et al. (2019), that the maxilla of DMNH EPV.96000 does not contribute to the margin of the foramen orbito-nasale, which is instead framed only by the prefrontal and palatine. Also in the palate, we do not observe a palatine-jugal contact, contrary to the original description (Lyson et al., 2019). Our CT data also allow us to unambiguously identify epipterygoids in DMNH EPV.96000, which were not identified as such in Lyson et al. (2019), although these authors suggested their tentative presence based on the fossa cartilaginis epipterygoidei, which holds the cartilaginous posterior part of the epipterygoid even in turtles in which the bone is ossified (e.g., Gaffney, 1979a). Epipterygoids have sometimes been found in material for which it was originally not described (see, for instance, also Spicher et al. 2023). Thus, the commonly inferred absence of epipterygoids among baenids should possibly be re-evaluated for a broader taxon sample with CT data. Contrary to the original description (Lyson et al., 2019), the occipital condyle of DMNH EPV.96000 is exclusively formed by the basioccipital, without exoccipital contributions. Lastly, 3D models of the mandible show that a dentary-articular contact is absent, although it was tentatively identified by Lyson et al. (2019).

The CT data also allowed us to extract new information regarding the cervical vertebrae. Unfortunately, only the axis is well preserved in DMNH EPV.96000. Nevertheless, its deeply concave posterior articulation surface clearly shows that the articulations surfaces of the cervical vertebrae of *Saxochelys gilberti* were at least formed (i.e., not platycoelous) in some cervical vertebrae, which is the derived condition among turtles (e.g., Joyce, 2007). On the other hand, *Saxochelys gilberti* shows the plesiomorphic presence of well-developed cervical ribs. These are smaller and less robust than in early stem turtles (e.g., *Proganochelys quenstedtii*: Gaffney, 1990; *Meiolania platyceps*: Gaffney, 1990, 1996) but are situated on equally robust, laterally projecting transverse processes that are broad and positioned centrally on the vertebrae. These features generally agree with those of *Boremys pulchra*, the only other baenid for which good vertebral material is known and described (Brinkman & Nicholls, 1991; Joyce & Lyson, 2015). Formed articulations and strong transverse processes can also be seen in cervical vertebral material of *Chisternon undatum* (Hay, 1908; Werneburg et al., 2015). Thus, the little available material of baenid cervical vertebrae seems to show a mosaic of plesiomorphic and derived vertebral features that is possibly consistent with their phylogenetic position as crownwardly-placed stem turtles (e.g., Evers & Benson, 2019; Joyce et al., 2016; Zhou & Rabi, 2015).

### High intraspecific variation in shell scute patterns

Lyson et al. (2019) already discuss that the shell of *Saxochelys gilberti*, which is known from more than 30 shells, is subject to high intraspecific variation. Lyson et al. (2019) specifically noted shape variations (e.g., size and form of plastral lobes, curvature vs. flatness of plastral surface), adult size variation, variation in the presence of supernumery bones, and variation in the sinuosity of some scute patterns. Our new observations on the holotype shell of *Saxochelys gilberti* add to this list, by showing that the omega-shaped femoral-anal sulcus of the plastral scutes does occur alongside specimens in which this feature is absent. Similarly, the extragular-humeral sulcus also shows variation involving the presence vs. absence of an omega-shaped contact. Plastral scute patterns are frequently used as phylogenetic characters in baenid studies and turtle studies more widely (e.g., Evers & Benson, 2019; Joyce et al., 2016; Rollot et al., 2022a, 2022b; Spicher et al., 2023; Zhou & Rabi, 2015), and also important for baenid taxonomy (Joyce & Lyson, 2015). Given that many fossils are only known from single specimens which cannot record the type of intrapecific variation that is apparent from the large sample of *Saxochelys gilberti* specimens, it is important to study the impact of polymorphic characters and more generally intraspecific variation. For turtles specifically, a recent study on geoemydids did not come to a clear solution on how to treat polymorphic characters in a phylogenetic analysis (Garbin et al., 2018). Until clear strategies are developed, we recommend to carefully document variational features. Lyson et al. (2019) identified some of the variable features in *Saxochelys gilberti* as potential sexual dimorphic traits, particularly plastral curvature and overall shell size. As the holotype specimen of *Saxochelys gilberti* is large (more than 29 cm) and has a flat plastral surface, we interpret the specimen to be a female, following Lyson et al. (2019).

## Conclusion

Our new segmentation models document the cranial and mandibular anatomy of *Saxochelys gilberti* in 3D. Together with the known postcranial material, which includes shell and non-shell material and the new referral of an associated shell to the holotype cranium, this makes *Saxochelys gilberti* one of the most complete and comprehensively documented species of baenid turtle, showing high levels of intraspecific variation in a number of shell features, including the shape of plastral sulci.

### Supplementary Information

**Additional file 1:** Phylogenetic matrix and character list as Nexus file.

## Data Availability

The CT data and derivative 3D models are deposited at MorphoSource under Project ID 000456859. The project can be assessed under the following link: https://www.morphosource.org/projects/000456859. The specimen DMNH EPV.96000 itself is deposited at the Denver Museum of Nature & Science and available for study under the local curator’s discretion. The phylogenetic matrix used in this study is available in this published article and its supplementary information files.
